# Find the right sample: A study on the versatility of saliva and urine samples for the diagnosis of emerging viruses

**DOI:** 10.1186/s12879-018-3611-x

**Published:** 2018-12-29

**Authors:** Matthias Niedrig, Pranav Patel, Ahmed Abd El Wahed, Regina Schädler, Sergio Yactayo

**Affiliations:** 1 Robert Koch Institute, Berlin, Germany; 2 TIB MOLBIOL Syntheselabor GmbH, Berlin, Germany; 3 Division of Microbiology and Animal Hygiene, University of Goettingen, Goettingen, Germany; 4 Control of Epidemic Diseases (CED), World Health Organization, Geneva, Switzerland

**Keywords:** Sample type, Emerging diseases, Viruses, Diagnostics

## Abstract

**Background:**

The emergence of different viral infections during the last decades like dengue, West Nile, SARS, chikungunya, MERS-CoV, Ebola, Zika and Yellow Fever raised some questions on quickness and reliability of laboratory diagnostic tests for verification of suspected cases. Since sampling of blood requires medically trained personal and comprises some risks for the patient as well as for the health care personal, the sampling by non-invasive methods (e.g. saliva and/ or urine) might be a very valuable alternative for investigating a diseased patient.

**Main body:**

To analyse the usefulness of alternative non­invasive samples for the diagnosis of emerging infectious viral diseases, a literature search was performed on PubMed for alternative sampling for these viral infections. In total, 711 papers of potential relevance were found, of which we have included 128 in this review.

**Conclusions:**

Considering the experience using non-invasive sampling for the diagnostic of emerging viral diseases, it seems important to perform an investigation using alternative samples for routine diagnostics. Moreover, during an outbreak situation, evaluation of appropriate sampling and further processing for laboratory analysis on various diagnostic platforms are very crucial. This will help to achieve optimal diagnostic results for a good and reliable case identification.

**Electronic supplementary material:**

The online version of this article (10.1186/s12879-018-3611-x) contains supplementary material, which is available to authorized users.

## Background

The emergence of several virus infections during the last decades like dengue, West Nile (WN), Severe acute respiratory syndrome (SARS), chikungunya (CHIK), Middle East respiratory syndrome coronavirus (MERS-CoV), Ebola (EBO), Zika (ZIK) and Yellow Fever (YF) raised some questions on quickness and reliability of laboratory diagnostic tests for verification of suspected cases. All important questions arising with an emerging infection like source and route of transmission, infectivity of patients, recommendation of safety measures for health care personal and distribution in an animal reservoir or other vectors require the performance of laboratory diagnosis. To investigate the patient’s infections most often a blood sample is taken not only for the analysis of blood parameters but also for pathogen detection mostly by PCR or for testing the presence of specific antibodies. However, sampling of blood requires medically trained personal and comprises some risks for the patient as well as for the health care personal. In particular the blood sampling of patients with highly infectious haemorrhagic fever like Ebola involve a great risk for the physician and the patient. Therefore, the sampling by non-invasive methods (e.g. saliva and/ or urine) might be a very valuable alternative for investigating a diseased patient. The use of saliva and urine for the diagnosis for many viral infections has been reported, however, it is often not performed in the broad routine diagnostics.

After a viral infection it usually takes some days until the propagation of the virus causes symptoms and a clinical investigation of a patient is indicated. Blood sampling for analysing blood parameters belongs to a routine procedure in developed countries. This is different in developing countries, where extensive analyses of clinical parameters are not performed routinely due to financial and technical constraints. Even though the sampling of swaps from the respiratory tracks is a common procedure for influenza, SARS, MERS-CoV and other respiratory infections, the sampling of blood is the prevalent procedure for all other recently emerged viruses. However, with the introduction of the highly sensitive Nucleic Acid Tests (NAT) for diagnostic also additional samples like saliva and/or urine were included in diagnostic sample collections. The advantage of using non-invasive samples like saliva for dengue serology was successfully proven 18 years ago by Cuzzubbo et al. who discovered that dengue primary and secondary infections can be distinguished by the level of IgG in saliva [1]. The specificity of the diagnosis of acute flavivirus infections like dengue, WN and YF has been improved by applying the NAT because it is not impeded by the widespread cross-reactivity found for most serological assays [2]. Since urine, saliva and other non-invasive samples were not part of the routine investigation, the sampling procedures and the samples containment are also very different. These differences complicate the interpretation of diagnostic findings and therefore an optimization for these samplings is required. An improvement of the sampling and stabilization of viral RNA in the diagnostic material will help to increase significantly the diagnostic sensitivity.

To analyse the usefulness of alternative non-invasive samples for diagnostic of infectious viral diseases like SARS, WN, dengue, MERS-CoV, Ebola, Zika and YF, we reviewed the importance of saliva and urine for the diagnosis of recently emerging viruses in comparison to conventional blood sampling. Since the extensive evaluation of different samples for human patients is often not performed, also investigations of animal models or vectors are considered. Therefore, we performed an electronic literature review on PubMed for alternative sampling for SARS, WN, dengue, MERS-CoV, Ebola, Zika and YF.

## Main text

### Method of literature search

A review of the literature for urine and saliva samples for diagnostic of CHIK, dengue, Ebola, WN, Zika, YF, SARS and MERS was performed using the electronic database PubMed. The period for searches was 1^st^ January 1980 – 26^th^ August 2016.

Appropriate articles were selected according to their abstracts and further reviewed for reason for diagnostic analysis, sample types used and the methods applied for diagnosis focusing on saliva and urine as test samples.

Search profile used for this review in PubMed for CHIK as an example:

"chikungunya virus"[MeSH Terms] OR ("chikungunya"[All Fields] AND "virus"[All Fields]) OR "chikungunya virus"[All Fields] AND ("saliva"[MeSH Terms] OR "saliva"[All Fields])

"chikungunya virus"[MeSH Terms] OR ("chikungunya"[All Fields] AND "virus"[All Fields]) OR "chikungunya virus"[All Fields] AND ("urine"[Subheading] OR "urine"[All Fields] OR "urine"[MeSH Terms])

Another 42 references were considered for analysing the preparation and storage of diagnostic samples as demonstrated in Table 1, see Additional file 1 for detailed methodology.

**Table 1:** Overview of selected publications describing samples used for viral diagnostic

Type of specimens		Arboviruses					VHFV	Coronaviruses	
		CHIKV	DENV	WNV	YFV	ZIKV	EBOV	SARS	MERS
Blood derived samples	whole blood	[139]	[143]	[147, 148]	n.r.	[88]	[160]	[165]	[170]
	peripheral blood (cells)	[139]	[144]	[147, 148]	n.r.	n.r.	[54]	[166]	
	peripheral blood (plasma)	[140]	[13]	[149]	[152]	[89]	[54]	[112, 166]	
	serum^a^	[141]	[144]	[149]	[152]	[97]	[52]	[167]	[169]
	umbilical cord blood	n.r.	n.r.	n.r.	n.r.	n.r.	n.r.	n.r.	n.r.
Body fluids	saliva	[19]	[13]	n.r.	?	[88, 89, 97]	[52]	n.r.	n.r.
	urine	[19]	[19]	[75]	[104]	[88, 89, 97]	[54]	[110, 112]	[124]

*VHF* Viral haemorrhagic Fever, ^a^ = NAT & Immunological test, *n.r.* not reported

The literature research revealed 711 papers, of which we considered 128 in this study (Additional file 1: Figure S1). Furthermore 43 publication analysing the sample processing and preparation were included.

Our analysis shows that there should be put more emphasis on non-invasive sampling to disclose more easy alternatives for collecting human samples for clinical and routine investigations. The present review provides some indications that non-invasive sampling offers a promising alternative for routine diagnostic as well as a favourable diagnostic approach for surveillance studies with high numbers of samples. The review provides an overview of the usability of non-invasive sample for diagnostics of emerging viruses and tries to give recommendation on how this experience could be helpful for future possible outbreaks of emerging viruses.

### Chikungunya

After an epidemic at La Reunion in 2005, an autochthone outbreak in Italy in 2007 and, spread in Caribbean islands in December 2013, chikungunya (CHIK) emerged in 2014 for the first time in Central America spreading into South America and North America [3–7]. Typical clinical specimens for CHIKV are blood, serum and plasma, but also urine, saliva and in rare cases CSF (Table 1, Additional file 1: Table S1). A systematic evaluation of the CHIK infection in mice and Cynomolgus macaques revealed the infectivity of different body fluids like saliva, urine and vaginal swaps [8]. This could be confirmed by analysis of saliva samples from 4 out of 13 acute CHIKV patients with haemorrhagic manifestations, which were found to contain viral RNA and infectious virus. Another study shows that RNA of chikungunya virus can be detected in the saliva at the first seven days of infection but with a lower efficacy than blood samples [9]. Also the detection of CHIKV genome by RT-PCR in urine was successful in one returning traveller, which is a valuable confirmation of the positive serology [10]. Based on the experience with other viral infections, the analysis of throat swabs and urine are part of recommendation for CHIK diagnostic [11].

### Dengue

Common clinical specimens used for dengue infection are blood, plasma and serum, but also in some cases CSF and breast milk (Table 1, Additional file 1: Table S2). Interestingly, the twenty-two selected publications described very well the progress of using saliva and urine as non- invasive samples. Based on the existing IgG ELISA for serology, they found that the IgG levels in saliva could be applied to differentiate between dengue primary and secondary infection [1]. One year later, Artimos de Oliveira et al., used saliva for detection of specific IgM of recent infections [12]. In 2000 Torres et al., found for the first time evidence of DENV RNA in saliva and plasma by RT-PCR [13]. The evaluation of saliva samples as alternative approaches for serological diagnosis (IgM, IgG, IgA) was further investigated and complemented by more systematic kinetics analysis of 1^st^ and 2^nd^ infections also comprising urine as an additional alternative sample [14, 15]. Since the sensitivity (93.3%), specificity (100%), positive predictive value (100%), and negative predictive value (83.3%) of the detection of IgG in saliva are high, it was considered as promising sample type for dengue diagnostics [16]. Also in case reports analysing acute infections, the detection of DENV in urine and saliva by RT-PCR as well as the application of filter-paper for saliva sampling prove the usefulness of these non-invasive samples [17–19]. The utility of saliva in an assay that detects DENV-specific IgA in the early phase of a 2^nd^ dengue infection shows 100% sensitivity from day-one after fever onset with a good correlation to IgA levels in serum and 97% specificity [20]. DEN NS1 antigen was detected in urine in around two third of dengue fever and DEN haemorrhagic fever (HF) using ELISA. Follow up the excretion of NS1 in urine is very important particularly in patients developing DF or DHF as an indication of protein leakage. The study demonstrates that the NS1 strip assay is less sensitive for urine compared to the ELISA and both assays need further adaptation to improve the sensitivity [21]. This was confirmed by a later study from Saito et al. showing that urine samples could be used for NS1 antigen ELISA but only in case of serum samples are very limited or in poor resource settings [22].

From 2012, more studies were performed using saliva and urine samples besides serum for quantitative RT-PCR and other parameters (NS1, IgM, IgG) for dengue diagnostic during the course of the disease [23–25]. Further studies underline the benefit of saliva as a diagnostic tool for use in developing tropical countries like India nevertheless further research is encouraged before implementation [26]. The delayed release of DENV in urine allows the diagnosis of an acute dengue infection by RT-PCR and sequencing on day 8 and 18, while the respective serum samples collected on the same period were negative for DENV [27].

All these promising results using urine and saliva samples for diagnostics stimulated further investigations on DENV RNA stability in urine and serum as well as establishing novel diagnostic device (stacking flow platform) for single-step detection of a target antibody in salivary fluid [28, 29]. Both studies investigated different conditions using urine or saliva samples for the diagnostics. One study focused on the storage conditions for these samples while the other improved the sample processing to optimize diagnostics using saliva. Andries et al. performed a representative study by analysing plasma, saliva and urine in 267 patients by applying various molecular and serological analyses. Unfortunately, the performances of these assays were better upon using plasma samples [30]. Analysing commercial rapid diagnostic tests (RDT) for detection of NS1/IgG/IgA in urine and saliva samples suggested that these RDT kits are of higher specificity and limited sensitivity [31]. Interestingly, in dengue secondary infection, the IgA in urine at 4-7 days of disease onset is higher than those with 1^st^ infections indicating severe Dengue [32]. Therefore, DENV IgA in urine is an indicator for the disease severity and must be investigated.

### Ebola and other viral hemorrhagic fevers (VHFs)

Ebola virus and other viral hemorrhagic fevers are mostly detected in blood derived samples such as whole blood, plasma or serum. Recent studies reported Ebola detection also in body fluids including saliva, urine, sweat, tears, semen and vaginal discharge as well as feces, ammonitic fluids, placenta and breast milk (Table 1 and Additional file 1: Table S3).The search for alternative sampling for Ebola yielded 34 publications from which 11 were performed during the recent Ebola outbreak from 2015/2016. Other publication were found for other VHF viruses like Hanta, 9 hits; Marburg, 4; Junin, 3; VHFs in general 2; Lassa, 2; Crimean Congo hemorrhagic fever (CCHF), 2; Rift Valley fever (RVF), 1; Guanarito, 1 and 1 for a novel Bunya virus.The first report dated from 1980 for RVF virus detection in saliva based on experimentally infected sheep and showed successful virus isolation from saliva [33]. This was completed by the extensive analysis of infected rodents as main transmission vectors for Hantavirus [34]. Besides virus excretion, Hanta virus could be demonstrated in several organs (lung, kidney, salivary glands and liver) by virus isolation from saliva, urine and feces for up to one year post inoculation. Also the intensive investigation of an imported Lassa case to the UK demonstrated the high viral load in blood taken eleven days post onset of the disease as well as significant virus count in urine on day 36, while throat swabs did not yield any detectable virus [35]. These findings were confirmed during the intensive analysis of an acute Lassa virus infected patient when the use of urine as additional sample to serum was successfully investigated for viral RNA by PCR [36].

Vereta and colleagues used urine and urine sediment epithelium for the identification of acute Hanta virus patients showing hemorrhagic fever with renal syndrome (HFRS) [37, 38]. Urine becomes an important sample for serological studies since antibody excretion in the urine coincided with the period of renal structure damage and stopped when the normal renal function was restored. The first successful Hantavirus isolation of a fatal HFRS case used urine and brain tissue and was further confirmed by RT-PCR [39]. Henceforward, urine was used for antigen and genome detection as well as for serology in experimentally infected pigs demonstrating that pigs can be the host for the viral transmission of HFRS virus [40]. Petterson et al. could demonstrate that hantavirus RNA was found in saliva few days after disease onset [41]. The association between the anti-Hantavirus IgA and Hantavirus RNA in saliva was inverse, the same is true for detecting RNA and antigen in endothelial cells within the parotid gland of HFRS patients [42]. In a recent study, the anti-Hantavirus antibodies in saliva was detected in a risk group in farms in Yorkshire, UK [43]. Hereby, the non-invasive saliva sampling confirms the usefulness of Hantavirus surveillance studies for analyzing the presence of Hantavirus in previously unknown risk areas. Also a recent investigation of Hanta virus persistence in wild rodents is based on the analysis of viral RNA in saliva, urine and feces show a peak during the first month after seroconversion continuing throughout the 8 month study [44].

Urine and saliva as non-invasive samples were also successfully used for analyzing the pathogenesis of Junin virus in the rodent vector responsible for virus transmission for up to 480 days post infection [45, 46]. Surviving animals showed a viral dissemination in brain, spleen, kidney and salivary glands with half of the animals shedding Junin virus and the other half being negative for infectious virus, while seroconverted. These findings were successfully used for analyzing the prevalence of Junin virus in the rodent population in endemic areas defining risk areas for the Junin virus infection [47].

Non-invasive samples like urine were used for the analysis of guinea pig and Rhesus macaques infectivity experiments with highly infectious and deadly Marburg virus [48]. In particular for dangerous biosafety level 4 pathogens the sampling of blood is very risky and laboratory infections are possible, which can be avoided by sampling urine. In the guinea pig model, the persistent shedding of Marburg virus in saliva, urine and feces showed that as early as by the end of incubation period and throughout the disease, the virus could be found in the feces and saliva virtually in the same concentrations [49], while in the blood the content of the virus was high and increased by the end of the disease. A similar approach was used investigating bat fecal and urine samples for Marburg virus after infection of a tourist visiting a cave in Uganda [50]. Around 2.5% of liver/spleen tissues of captured bats were positive in RT-PCR for Marburg virus. Moreover, the virus was detected in 15 different tissues and plasma of Egyptian fruit bats subcutaneously inoculated with Marburg virus, while limited results were obtained applying mucosal swab samples, urine and fecal samples [51].

Antibodies against Ebola virus could not be found in oral fluids, while 100% agreement between the presence of RNA in serum and oral fluids by RT-PCR was observed [52]. Moreover, in 26 confirmed Ebola cases, 16 samples including saliva, stool, semen, breast milk, tears, nasal blood and a skin swab were positive in Ebola culture and/or RT-PCR [53]. In case of EBOV, all samples including body fluids must be handled applying the WHO recommendations, since they are highly infectious.The recent Ebolavirus outbreak in Serra Leone, Liberia und Guinea with 11,325 deaths initiated several intensive investigations of different patient specimens like blood, urine, sweat, saliva, conjunctival swaps, stool and semen for infectious virus [54, 55]. These became a major interest since transmission routes were not always obvious from previous experience. Moreover, public health measures require clear references, when recovered patients are not infectious anymore and can be released from quarantine. The excretion of EBOV in body fluids from infected or recovered patients create a great risk for person in contact [54, 56] as the Ebola virus RNA was detected in the following body fluids weeks up to several months after infection: saliva, conjunctiva/tears, stool, vaginal fluid, sweat, urine, amniotic fluid, aqueous humor, cerebrospinal fluid, breast milk, and semen raised many concerns regarding the long term transmissibility of infectious virus causing a recrudesce of an outbreak [56–58]. Comparing whole-blood with urine specimen’s analysis from Ebola patients in a novel diagnostic film-array with RT-PCR shows a reasonable match of 90% for whole blood and 85% for urine [59]. Testing the stability of Ebola virus RNA in human blood and urine under environmental African conditions shows that viral RNA testing from blood samples stored in EDTA buffer is more sensitive [60]. Viral RNA in urine seems less stable compared to blood since urine samples were found positive by RT-PCR by day 10-14 compared to blood until at least day-18. Since the CCHF outbreak in Turkey in 2002, the detection of virus in other body fluids received attention to avoid secondary infections. RNA of CCHF virus was identified in the saliva and the urine of 5/6 and 2/3 patients, respectively [61]. This was confirmed by a later study, which detected CCHF virus in urine samples of patients with prolonged viremia [62].

Furthermore, nasopharyngeal aspirates and/or urine were considered as diagnostic samples evaluating a multiplex hybridization array for detecting various infectious diseases [63]. Interestingly, the Plasmodium falciparum was identified as a cause of death in a case of hemorrhagic fever like illness during the Marburg hemorrhagic fever outbreak in Angola in 2004-2005. Also a novel diagnostic array based on beads technology for detection of multiple bat-borne viruses including Ebola and Nipha viruses used urine from wild bats from Australia and Bangladesh for a surveillance study [64].

Analyzing cases in China of a hemorrhagic fever due to a novel Bunya virus, the RNA was identifed in the blood as well as in urine, throat, and fecal specimens [65]. Further studies are necessary to investigate the significance of non-invasive samples for analyzing the virus pathogenesis and the infectivity of different body fluids.

### West Nile

Clinical specimens used for WNV detection are blood derived samples and CSF (Table 1 and Additional file 1: Table S4). Since the safety of blood banks was important, testing of blood samples was the evident choice. Even though Steele et al. found infectious WN in several organs (brain, heart, spleen, liver, kidney, adrenal intestines, pancreas, lung and ovary) of birds, it took four years until Tesh and others published the investigation of urine samples used for pathogenesis studies in hamsters and birds [66–69]. These findings were later confirmed in different animal models like chipmunks, fox squirrels, hamsters and mice analyzed for WNV pathogenesis research [70–74]. The first report of WN detection in urine was in an acute severe case with encephalitis found positive eight days after symptoms onset [75]. Further studies detected WNV RNA up to 6.7 years in urine indicating for a persistent renal infection for several years [76]. This was confirmed by a number of studies demonstrating the long persistence of WNV particularly in patients with WN fever compared to patients with WN neuro-invasive disease, which had a higher vireamia for a shorter period than those with West Nile fever [77–80]. In the meantime, the advantage of using urine instead or additionally to serum is demonstrated by many investigations and could prove its value also for routine diagnostic [81–86]. Also WNV could be successfully isolated from urine samples and further analyzed by sequencing.

In summary, one could state that the use of urine is a suitable alternative diagnostic sample compared to serum and should be listed in fact sheets from national and international organizations (WHO, PAHO, CDC, ECDC). Further studies are needed investigating if urine samples are a useful source for surveillance studies for WNV distribution and for analyzing the severity of the disease in patients with neuro-invasive and/or kidney manifestation.

### Zika

The Zika virus was detected in blood, serum, saliva, and urine, as well as in semen, vaginal discharge, sweat, tears, ammonitic fluids, placenta and breast milk (Table 1 and Additional file 1: Table S5). The expansion of Zika to Brazil *via* New Caledonia 2015 stimulates the development of diagnostic assays for NAT and serology to analyze the increasing number of cases [87]. In a surveillance study, ZIKV was identified in 19.2% (total 182 patients) of saliva samples, but not in blood [88]. The detection of ZIKV in saliva samples increased the molecular detection rate of ZIKV in acute case but ZIKV did not persist for longer time frame in saliva as in urine or semen. Nevertheless, saliva sample was advantageous in children and neonates [89]. Interestingly, the ZIKV can be detected in urine samples for even longer. Combination of urine and saliva besides serum is of great significant in ZIKV diagnostics [90]. This was also true in a rhesus macaque model used for analyzing plasma, saliva, urine and cerebrospinal fluid by RT-PCR for ZIKV detection [91].

Moreover, RNA of ZIKV was detected in semen by RT-PCR on the 7^th^ day of the appearance of symptoms but was not identified on the 21^st^ day, which was confirmed by Reusken et al. [92, 93]. This finding consequently leads to the possibility of an infection *via* sexual intercourse as documented by D'Ortenzio et al. with a high viral load in the semen obtained on sample collected after 18 days [94, 95]. In several smaller and bigger studies, the usability of saliva and urine beside blood samples was demonstrated for the Zika virus diagnostics [96–98]. The excretion of viral RNA in urine and saliva was observed for up to 29 days after symptom onset and with higher viral load than in blood [99, 100]. The high risk for transmission by patient’s body fluids also raised concern for appropriate safety measures for health care personal as discussed in several publications [101–103].

### Yellow Fever

Yellow Fever is generally detected in blood, plasma, serum and CSF as other flaviviruses (Table 1 and Additional file 1: Table S6). Although alternative samples demonstrate their usefulness for other flavivirus infections like urine or saliva samples for diagnostics of dengue or West Nile, YF were not investigated until 2011 [104, 105]. These are the first reports showing the usefulness of urine samples for diagnostic of adverse events after YF vaccination. For vaccinees developing severe side effects after vaccination it could be clearly shown that viral shedding is prolonged up to day 25 post vaccination. The release of YF RNA could be found in urine at day 198 after vaccination. If the detection of the YF attenuated 17D vaccine virus could be demonstrated in urine easily. However, representative data of alternative samples like urine or saliva from YF infections are missing. In particular for cases with a severe course of disease with numerous hemorrhagic bleedings the use of non-invasive sampling would be a real alternative preventing bodily injury of the patient.

### Severe acute respiratory syndrome (SARS)

When SARS emerged in November 2002, it was obvious to analyze samples from the respiratory tract for diagnostic. However, even for the first SARS patients other samples beside blood and nasopharyngeal aspirate samples like feces and urine were found positive with 97% and 42% for viral RNA, respectively [106] (Table 1 and Additional file 1: Table S7). This was confirmed by analysis of throat wash and saliva showing a high virus load up to 6x10^6^ and 6x10^8^ RNA copies per ml, respectively [107]. Moreover, the detection of SARS in plasma, sputum, endotracheal aspirates, stool, throat swabs and saliva revealed significant differences between the types of samples [108] as all samples from the lower respiratory tract were tested positive. Cheng et al. found that death was associated with higher virus load in nasopharyngeal specimens obtained on day 10 after the onset of symptoms [109]. Different samples like serum, nasopharyngeal aspirates (NPA), throat swabs, nasal swabs, rectal swab, stool and urine were used for analysis of the viral pathogenesis during the course of the disease [109–112]. The diarrhea and hepatic dysfunction was associated high viral load in NPA, while in urine is associated with abnormal urinalysis findings. The extensive analysis of 415 SARS patients revealed the presence of SARS in respiratory specimens two weeks after disease onset, and in stool, rectal swab and urine specimens for up to four weeks. Throat and nasal swabs is less significant that the NPA specimens, therefore, the high viral load in NPA has a major impact on the airborne transmission, which played a major role during the outbreak in Hong Kong [113].

The evaluations of commercial PCR kits and in-house antigen-ELISAs for the quality of SARS diagnostic are an important task to select the most appropriate test for the selected sample. The sensitivity of RT-PCR is higher compared to two antigen-ELISAs [114] as RNA of SARS-CoV can be detected earlier in fecal specimens in around 80% of patients and in 25% of urine samples. Based on all these findings, RT-PCR is the method of choice for early diagnosis of SARS CoV infection [115, 116].

The co-presence of viral RNA and antibodies in plasma was observed over three weeks in two cases [117]. In addition, the SARS-CoV was isolated from stool or urine specimens for longer than 4 weeks [118]. Since the detection of SARS virus is possible in various clinical samples (NPA, throat-swab, fecal, cerebrospinal fluid, blood and urine) by viral culture or RT-PCR clear and significant recommendations for diagnostic of SARS based on the previous experiments worked out by the WHO and the Multi-Centre Collaborative Network require an appropriate update. Even though SARS did not appear any more after the outbreak except for the two laboratory infections thereafter [119–171].

Bats are the most likely animal vector for CoVs [121]. Also experiments in mice and rhesus macaque analyzing the viral pathogenesis for the development of effective strategies for diagnosis, prevention, therapy and vaccine design was further evaluated [122, 123]. Since salivary gland epithelial cells were affected as a first target after infection the immunization with virus like particles might be eliciting a protective immune response against SARS-CoV generating a mucosal immunity. This new vaccine approach provides important information for future vaccine design.

### Middle East respiratory syndrome coronavirus (MERS-CoV)

A boost of nearly 50 publications on alternative sampling within three years demonstrates the importance and documents the public health concern with this respiratory disease caused by a new Corona virus (CoV) (Additional file 1: Table S8). The publications can be roughly categorized as follows 11 case reports, 7 descriptions of pathogenesis, 11 on recommendation and investigation of diagnostics, 7 on recommendation and analysis of risks for health care workers, 2 recommendations for patient management and 5 on control and prevention including treatment and vaccine development. Only a few selected publications were considered for this review. Bronchoalveolar and lower respiratory tract fluids were highly positive for MERS-CoV in the two identified cases [124], while urine samples were only positive on day 13. Stool samples and oro-nasal swabs contained very low MERS-CoV copy number on days 12 and 16, respectively, and no virus was detected in blood. In the beginning of the outbreak the way of transmission was rather unclear. Therefore, the virus replication in the kidney with potential shedding in urine was further investigated [125]. To clarify the different transmission routes a prediction model for oral-fecal and/or oral-saliva transmission routes for MERS-CoV was developed and evaluated [126]. Very soon camels as contributing animal host for MERS-CoV infection got into focus of investigation and it became obvious that also dromedary camels in Eastern Africa and the Arabian Peninsula have a very high seroprevalence of MERS-CoV antibodies [127]. For further surveillance studies in camels, hedgehogs, and bats very often non-invasive samples like urine, saliva, fecal nasal swaps, fecal swaps or camel milk were used to analyze for MERS-CoV RNA or specific antibodies [128]. In summer 2015, 183 confirmed cases of MERS and 33 fatalities were reported in the Republic of Korea caused by a nosocomial outbreak [129].

The timing and intensity of respiratory viral shedding in MERS-CoV infected patients is still of major interest as airborne transmission from human to human by droplets seems to be the dominant infection route [130–132]. It might be that the introduction of the isothermal amplification (LAMP or RPA) for detecting of emerging diseases provides a useful tool for quick and reliable diagnostic of acute cases and might lead to an immediate public health response including the appropriate safety precautions for the medical personal [133, 134].

Based on the experience with other respiratory infections and based on the lessons learned from the previous SARS outbreak the analysis of respiratory samples are parts of the routine samples taken for diagnostic of suspected MERS patients.

## Conclusions

The analysis of using other non-invasive samples for virus diagnostics clearly shows that the procedures for the optimal sampling strategy of the different viral infections are not seriously investigated table S9. In the following overview it became obvious that for the newly emerging viruses like CHIK, MersCoV and Zika more and broader attempts for sampling strategies were performed and investigated than for well known viruses like YF and dengue.

The application of saliva as a sample of choice for the diagnosis of CHIK fever infections in small children or patients whose blood samples are difficult to obtain is of great significant. However, all recommendations/fact sheets for CHIK from national and international organizations (CDC, PAHO, WHO, ECDC) do not mention saliva or urine samples as suitable material for diagnosis because of the missing of meaningful studies that evaluate saliva and urine as diagnostic samples compared with blood. Hopefully, there will be studies performed answering these questions with a representative number of CHIK patients in the future.

The use of saliva and urine for the diagnosis of acute dengue infections is of great importance and requires more attention. Present studies show the usefulness for these samples for diagnostics and also the monitoring of the severity of infection. In particular for surveillance studies and/or investigation of small children the noninvasive approach using saliva or urine for the laboratory diagnosis of dengue cases should be considered when blood samples are difficult to obtain. Although the application of alternative samples for the diagnostic became more popular in the recent years, further studies and improvements are necessary to establish these samples equally to common plasma or serum samples. Commercial assays as well as appropriate sampling equipment have to be verified for diagnostic applicability in representative cohorts and during the course of the disease. It seems that the increased awareness of alternative samples stimulates such studies for dengue diagnosis as demonstrated in the recent publications mentioned above.

In particular for diagnostic of VHF cases, the investigation of the usefulness of non-invasive taken samples should have high priority since blood sampling from such patients is highly risky and often dangerous for patients with hemorrhagic symptoms while continuous bleeding occurs. The virus load in sever cases of VHF is very high in body fluid and the NAT is the method of choice since the production of specific IgM and/or IgG antibodies takes a few days. In very severe cases, the patient even can pass away before developing a detectable antibody reaction, while a general viremia affecting several organs and is detectable in most body fluids. In less severe cases, the exact moment becomes an important issue since viremia only remains for just a few days (3-4 days) and often patient samples were taken too late for successful NAT thus requiring laborious serology testing with in-house assays and risk of false positive reactivity. Onset of disease is also not a well-defined point of time because the perception of a disease might be different in developing compared to developed countries. This makes the comparison of clinical symptoms and diagnostic findings during the course of the disease even more difficult.

Although the analysis of different body fluids during the recent Ebola virus outbreak expands our knowledge of virus transmissibility via eye fluid and semen tremendously, systematic studies on which and for how long the different body fluids will contain viral RNA and/or infectious viruses are still missing. Recommendations on how non-invasive sampling should be performed, what kind of samples are necessary and how they should be stabilized and transported under African environmental conditions are still missing and require thoroughly investigations.

For WNV infections the use of urine is a suitable alternative diagnostic sample compared to serum and should be listed in fact sheets from national and international organizations. Further studies are needed investigating if urine samples are a useful source for surveillance studies on WNV distribution and for analyzing the severity of the disease in patients with neuro-invasive and/or kidney manifestation. The emergence of Zika virus tremendously stimulated the investigation of using non-invasive samples as saliva and urine for diagnostic of Zika virus infections. From the start of the Zika outbreak in French Polynesia saliva was considered as alternative material for the diagnostics. The investigation of urine and saliva shows a higher virus load compared to blood making these samples a suitable alternative to blood. However, this information has to be forwarded to the physicians as it is already part of the PAHO and WHO recommendations.

For YF diagnostic one can hope that the integration of saliva and urine as alternative samples into the recommendation worked out by the WHO for the recent outbreak will help to implement this method into the medical procedure and create some meaningful results for future outbreak scenarios.

Even although SARS seems to have disappeared from the globe, the experience and findings on which and when the different diagnostic samples should be used for a quick and reliable diagnostic should be carefully evaluated, since respiratory infections are on the rise as we have seen a few years later by the influenza and MERS-CoV outbreaks. The great variety on which, how and when respiratory samples have to be taken is reflected by the list of nomenclature found in the publications: for upper respiratory specimens (nasal swab, exudate nose, oronasal swab, pharyngeal swab, tracheal swab, exudate mouth, saliva and sputum) and lower respiratory specimens (bronchoalveolar fluid, bronchoalveolar lavage). Recommendations for standardized procedures for sampling and handling of the respiratory samples will help to allow a better comparison of the findings and provide a better preparedness for the next respiratory disease outbreak.

Because of a higher virus load, samples from the lower respiratory tract are more suitable for diagnostic. However, the sampling of bronchoalveolar-lavage fluid is also invasive and requires an experienced physician and holds a certain risk of nosocomial infections by aerosols. Other non-invasive samples like urine, nasal, pharyngeal, tracheal and rectal swabs were included in the diagnostic analysis for analyzing the pathogenesis and the kinetic of virus release by the different body fluids. Like for SARS standardized procedures for sampling and handling of the respiratory samples will help to allow a better comparison of the findings and provide a better preparedness for future respiratory disease outbreak.

The present review clearly shows that there is still the need to perform more diligent studies analyzing non-invasive patient samples in comparison with blood and serum samples, which used for routine diagnostic of emerging viral diseases. While for some diseases like the respiratory infections, the sampling of nasal swaps and saliva is part of routine sampling, for other viral infections, blood or serum seems the predominant and only type of sample used for laboratory diagnosis.

This is in particular disappointing because non-invasive sampling offers a real alternative avoiding additional risk for the patient and the medical personal. Patients suspected to be infected by VHF usually have a very high virus load. Therefore, the analysis of saliva or urine should be routinely performed in parallel to investigation of blood samples. Based on a solid database it will be possible to determine the most appropriate sample for a viral diagnosis before it gets implemented in the routine diagnostic procedure. Diagnostic laboratories and physicians have to gain knowledge when and how alternative samples have to be considered for analyzing an acute or convalescent viral infection. Even though, we have with NAT a very sensitive diagnostic tool, the best point in time during the course of the disease together with the optimal sampling, sample storage and sample preparation are essential to get optimal results for a laboratory diagnosis. In patients presenting with a moderate or mild disease, the viremia is mostly very short lasting just 1-3 days. For these patients the positive NAT is sometimes difficult to achieve and a negative finding is of no predictive value since the virus titer might be just below the detection limit or sample storage and sample preparation is not optimal in case of non-invasive sampling such as saliva and urine. Although the virus disappeared from the blood, the virus genome will be still detectable in the urine as consequence of the clearance process as we have found in Yellow Fever vaccinees or other for Zika [97, 104]. In contrast, we find a high viral load in patients developing a severe disease sometimes with a general infection affecting several organs. In particular in those patients, the detection of the pathogenic virus by NAT is not difficult analyzing different types of samples. Very often the viral load also reflects the severity of the disease and can be used as prognostic marker for progression of the disease in the patient. Exactly for these patients the non-invasive sampling is less stressful as frequent bleeding performed with the current procedure. This becomes even more important, if point of care diagnostic allows a more frequent monitoring of infection parameters of the diseased patient in the future. For the recently emerging CHIK and Zika in South America, the analysis of throat swaps, saliva and urine samples were already analyzed for their usability in diagnostics. However, the findings are based on case studies or small studies and require a more systematic approach. Preferably there should be performed a systematic analysis of different invasive samples (blood, bronchoalveolar lavage) and non-invasive samples (saliva, urine, etc.) for their diagnostic value using different diagnostic methods for patients with acute and convalescent disease when new infections emerge. This also implies that the procedure taking samples has to be somehow standardized. This became very obvious by the large number of terms for upper and lower respiratory samples most likely reflecting the great variety how the samples were taken. This makes the comparability of diagnostic findings presented in different studies very difficult. To address this problem a clear recommendation for the sampling procedure would help to overcome this imponderability. Furthermore, the methods for storage of the samples regarding temperature and stabilization needs to be worked out to allow an optimal transport and preparation of the diagnostic sample. For example there are special sampling tubes with stabilizer available for both saliva (DNAgard Saliva by biomatrica, USA) and urine (VACUETTE Urine CCM tubes by Greiner Bio-One, Germany and Urine Monovette by Sarstedt, Germany), which will help preserves the integrity of sample at room temperature. Sample preparation from urine and saliva could be also challenging especially for molecular diagnostics, therefore it is very important to optimize protocols using clinical samples rather than spiked mock-samples. Our personal experience showed that the optimized sample preparation method on real clinical samples was not as efficient as on spiked mock samples.

The increasing number of publications considering alternative sampling for diagnostic of the recent outbreaks MERS-CoV, CHIK, Ebola and Zika are promising. However, systematic studies are rare and for some infectious disease outbreaks like the recent yellow fever in Angola, non-invasive samples for diagnostic are still uncommon and require further investigation. Just for diseases like yellow fever, non-invasive samples like urine or saliva might be a very practical alternative, since the first index patients are detected often very late, while blood will be negative by NAT in patients with mild disease and from patients with a severe disease it is difficult taking blood because of a hemorrhage clinical picture. Non-invasive samples might be also a great alternative for investigations of children or other patients were collection of blood samples is difficult [18, 30, 88]. Also for large surveillance studies, non-invasive samples like saliva and/or urine could be performed without medical personal for collecting blood. However, the usability of these samples for serology or NAT has to be evaluated in comparison to conventional use of sera. Therefore, it is necessary to introduce non-invasive sampling strategies in recommendations as done for the “Yellow Fever Laboratory Diagnostic Algorithm” worked out for the epidemic and non-epidemic countries in Africa during the YF outbreak in Angola by WHO expert team [135]. Since these investigations could be only performed during an outbreak situation the preparation of a scheme for evaluation of sampling for respiratory as well as for other infectious diseases including the further processing for laboratory diagnostic is highly recommended. This more structured approach will help to improve the diagnostic of emerging infectious diseases significantly as new diagnostic methods and platforms are developed continuously and require effective evaluation under real conditions.

For confirmation of a clinical diagnosis a correct laboratory diagnosis is essential for all further steps of patient's treatment and handling. In particular for new and/or emerging diseases, the conditions for the best sampling regarding time and source (blood, serum, saliva, urine, etc.) are often not known. Since every disease has its own course of symptoms and affected organs, the optimal sampling schema might change and require different strategies regarding sample preparation and processing. While in the early phase when disease symptoms appear often the viral pathogen could be detected by nucleic acid testing in blood or other body fluids in the later phase only antibodies are detectable in serum.

In the present article, we analyzed over 700 scientific publications on diagnostic approaches for dengue, West Nile, SARS, chikungunya, MERS-CoV, Ebola, Zika and Yellow Fever regarding their relevance for using alternative samples like saliva and urine for their versatility for virus diagnostic. The use of non-invasive samples offers a suitable alternative for improving the diagnostic performance and significantly reduces the risk for medical personal and patients for routine diagnostic and during an outbreak situation. The advantage of using non-invasive samples for the diagnostic has to be seriously analyzed and investigated before implementing in the routine diagnostics.

## Additional file

Additional file 1: **Figure S1.** Selection scheme flowchart. Abbreviation: CHIV = Chikungunya Virus, VHF = Viral Hemorrhagic Fever, WN = West Nile, YF = Yellow fever. **Table S1.** Analysis of different sampling methods for diagnostic of CHIK virus infection. **Table S2.** Analysis of different sampling methods for diagnostic of Dengue virus infection. **Table S3.** Analysis of different sampling methods for diagnostic of Ebola/VHF virus infections. **Table S4.** Analysis of different sampling methods for diagnostic of West Nile virus infection. **Table S5.** Analysis of different sampling methods for diagnostic of Zika virus infection. **Table S6.** Analysis of different sampling methods for diagnostic of Yellow Fever virus infection. **Table S7.** Analysis of different sampling methods for diagnostic of severe acute respiratory syndrome (SARS) virus infection. **Table S8.** Analysis of different sampling methods for diagnostic of Middle East respiratory syndrome coronavirus (MERS-CoV) infection. (DOCX 162 kb)

## Abbreviations

AG test: Antigen capture test
CCHF: Crimean Congo hemorrhagic fever
CDC: Centers for Disease Control and Prevention
CHIK: Chikungunya
CoV: Corona virus
CSF: Cerebrospinal fluid
DENV: Dengue virus
DF: Denge fever
DHF: Dengue hemorrhagic fever
EBOV: Ebola virus
ECDC: European Centre for Disease Prevention and Control
ELISA: Enzyme-linked immunosorbent assay
GAPDH: Glyceraldehyde 3-phosphate dehydrogenase
HAI: Hemagglutination inhibition assay
HF: Hemorrhagic fever
HFRS: Hemorrhagic fever with renal syndrome
IFA: Immunofluorescence assay
IgA: Immunoglobulin A
IgG: Immunoglobulin G
IgM: Immunoglobulin M
IH: Immune histochemistry
LAMP: Loop-mediated isothermal amplification
LRS: Lower respiratory specimens
MERS-CoV: Middle East respiratory syndrome coronavirus
NAT: Nucleic Acid Test
NPA: Nasopharyngeal aspirates
NS1: Nonstructural protein 1
PAHO: Panamerican Health Organisation
PCR: Polymerase Chain Reaction
PRNT: Plaque reduction and neutralization test
RDT: Rapid diagnostic test
RNA: Ribonucleic acid
RPA: Recombinase polymerase amplification
RT-PCR: Reverse transcriptase polymerase chain reaction
RVF: Rift Valley fever
SARS: Severe acute respiratory syndrome
TMA: Transcription-mediated amplification
URS: Upper respiratory speci¬mens
VHF: Viral hemorrhagic fever
VI: Virus isolation
WHO: World Health Organisation
WN: West Nile
WNV: West Nile virus
YF: Yellow fever
ZIKV: Zika virus
