# Supplementary material for: Find the right sample: A study on the versatility of saliva and urine samples for the diagnosis of emerging viruses
Source: BMC Infect Dis. 2018 Dec 29;18:707. doi: 10.1186/s12879-018-3611-x (PMC6311079; doi:10.1186/s12879-018-3611-x)
Supplement: Supplementary file 1 — Figure S1. Selection scheme flowchart. Abbreviation: CHIV = Chikungunya Virus, VHF = Viral Hemorrhagic Fever, WN = West Nile, YF = Yellow fever. Table S1. Analysis of different sampling methods for diagnostic of CHIK virus infection. Table S2. Analysis of different sampling methods for diagnostic of Dengue virus infection. Table S3. Analysis of different sampling methods for diagnostic of Ebola/VHF virus infections. Table S4. Analysis of different sampling methods for diagnostic of West Nile virus infection. Table S5. Analysis of different sampling methods for diagnostic of Zika virus infection. Table S6. Analysis of different sampling methods for diagnostic of Yellow Fever virus infection. Table S7. Analysis of different sampling methods for diagnostic of severe acute respiratory syndrome (SARS) virus infection. Table S8. Analysis of different sampling methods for diagnostic of Middle East respiratory syndrome coronavirus (MERS-CoV) infection. (DOCX 162 kb) [file 12879_2018_3611_MOESM1_ESM.docx]

**Supplement material**

**Find the right sample: A review of versatility of saliva and urine samples for the diagnosis of new emerging viruses.**

Matthias Niedrig^*^, Pranav Patel, Ahmed Abd El Wahed, Regina Schädler, Sergio Yactayo

* corresponding author

**Methodology**

Search profile used for this review in PubMed:

"chikungunya virus"[MeSH Terms] OR ("chikungunya"[All Fields] AND "virus"[All Fields]) OR "chikungunya virus"[All Fields] AND ("saliva"[MeSH Terms] OR "saliva"[All Fields])

"chikungunya virus"[MeSH Terms] OR ("chikungunya"[All Fields] AND "virus"[All Fields]) OR "chikungunya virus"[All Fields] AND ("urine"[Subheading] OR "urine"[All Fields] OR "urine"[MeSH Terms])

"dengue virus"[MeSH Terms] OR ("dengue"[All Fields] AND "virus"[All Fields]) OR "dengue virus"[All Fields] AND ("saliva"[MeSH Terms] OR "saliva"[All Fields])

"dengue virus"[MeSH Terms] OR ("dengue"[All Fields] AND "virus"[All Fields]) OR "dengue virus"[All Fields] AND ("urine"[Subheading] OR "urine"[All Fields] OR "urine"[MeSH Terms])

"ebolavirus"[MeSH Terms] OR "ebolavirus"[All Fields] OR ("ebola"[All Fields] AND "virus"[All Fields]) OR "ebola virus"[All Fields] AND ("urine"[Subheading] OR "urine"[All Fields] OR "urine"[MeSH Terms])

"ebolavirus"[MeSH Terms] OR "ebolavirus"[All Fields] OR ("ebola"[All Fields] AND "virus"[All Fields]) OR "ebola virus"[All Fields] AND ("saliva"[MeSH Terms] OR "saliva"[All Fields])

"west nile virus"[MeSH Terms] OR ("west"[All Fields] AND "nile"[All Fields] AND "virus"[All Fields]) OR "west nile virus"[All Fields] AND ("saliva"[MeSH Terms] OR "saliva"[All Fields])

"west nile virus"[MeSH Terms] OR ("west"[All Fields] AND "nile"[All Fields] AND "virus"[All Fields]) OR "west nile virus"[All Fields] AND ("urine"[Subheading] OR "urine"[All Fields] OR "urine"[MeSH Terms])

"zika virus"[MeSH Terms] OR ("zika"[All Fields] AND "virus"[All Fields]) OR "zika virus"[All Fields] AND ("urine"[Subheading] OR "urine"[All Fields] OR "urine"[MeSH Terms])

"zika virus"[MeSH Terms] OR ("zika"[All Fields] AND "virus"[All Fields]) OR "zika virus"[All Fields] AND ("saliva"[MeSH Terms] OR "saliva"[All Fields])

"yellow fever virus"[MeSH Terms] OR ("yellow"[All Fields] AND "fever"[All Fields] AND "virus"[All Fields]) OR "yellow fever virus"[All Fields] AND ("urine"[Subheading] OR "urine"[All Fields] OR "urine"[MeSH Terms])

"yellow fever virus"[MeSH Terms] OR ("yellow"[All Fields] AND "fever"[All Fields] AND "virus"[All Fields]) OR "yellow fever virus"[All Fields] AND ("saliva"[MeSH Terms] OR "saliva"[All Fields])

("sars virus"[MeSH Terms] OR ("sars"[All Fields] AND "virus"[All Fields]) OR "sars virus"[All Fields] OR ("sars"[All Fields] AND "coronavirus"[All Fields]) OR "sars coronavirus"[All Fields]) AND ("saliva"[MeSH Terms] OR "saliva"[All Fields])

("sars virus"[MeSH Terms] OR ("sars"[All Fields] AND "virus"[All Fields]) OR "sars virus"[All Fields] OR ("sars"[All Fields] AND "coronavirus"[All Fields]) OR "sars coronavirus"[All Fields]) AND ("urine"[Subheading] OR "urine"[All Fields] OR "urine"[MeSH Terms])

("middle east respiratory syndrome coronavirus"[MeSH Terms] OR ("middle"[All Fields] AND "east"[All Fields] AND "respiratory"[All Fields] AND "syndrome"[All Fields] AND "coronavirus"[All Fields]) OR "middle east respiratory syndrome coronavirus"[All Fields] OR ("mers"[All Fields] AND "cov"[All Fields]) OR "mers cov"[All Fields]) AND ("saliva"[MeSH Terms] OR "saliva"[All Fields])

("middle east respiratory syndrome coronavirus"[MeSH Terms] OR ("middle"[All Fields] AND "east"[All Fields] AND "respiratory"[All Fields] AND "syndrome"[All Fields] AND "coronavirus"[All Fields]) OR "middle east respiratory syndrome coronavirus"[All Fields] OR ("mers"[All Fields] AND "cov"[All Fields]) OR "mers cov"[All Fields]) AND ("urine"[Subheading] OR "urine"[All Fields] OR "urine"[MeSH Terms])

Figure S1: Selection scheme flowchart. Abbreviation: CHIV = Chikungunya Virus, VHF = Viral Hemorrhagic Fever, WN = West Nile,
 YF = Yellow fever

Additional records for
MERS-CoV identified through Scolar search (n = 48)

Records identified through database PubMed search

(n = 711)

Articles assessed for eligibility
 (n = 711)

Articles included in the evaluation
 (n = 128)

Dengue n = 22

YF
n = 2

MERS-CoV
n = 11

SARS-CoV
n = 17

Zika
n = 16

WN
n = 20

Ebola, VHF
n = 36

CHIKV
n = 4

SARS

saliva n = 12

urine n = 8

MERS-CoV

saliva n = 11

urine n = 15

YF

saliva n = 83

urine n = 78

Zika

saliva n = 27

urine n = 4

WN

saliva n = 39

urine n = 29

Ebola, VHF

saliva n = 101

urine n = 234

Dengue

saliva n = 83

urine n = 78

CHIK

saliva n = 36

urine n = 8

Total records screened by title/abstract
 saliva n = 320, urine, n = 391
(total n = 711)

**Table S1: Analysis of different sampling methods for diagnostic of CHIK virus infection**

| **sample type** | **diagnostic methods** | **reason for analysis** | **results, key findings** | **reference** |
| --- | --- | --- | --- | --- |
| oral fluid,  vaginal swaps,  urine,  saliva | RT-PCR,  VI | analysis of pathogenicity in mouse and monkeys analysis of human saliva for diagnostic | Infectious virus was present in the oral cavity of CHIKV infected mice, likely due to hemorrhagic lesions in the olfactory epithelium that allow egress of infected blood into the nasal, and subsequently, oral cavities. In addition, mice were more susceptible to infection with CHIKV via intranasal and oral routes, with mice also able to transmit virus mouse-to-mouse without an arthropod vector. Cynomolgus macaques often show bleeding gums after CHIKV infection, and analysis of saliva from several infected monkeys also revealed the presence of viral RNA and infectious virus.  Furthermore, saliva samples from 4 out of 13 acute CHIKV patients with hemorrhagic manifestations were found to contain viral RNA and infectious virus. Oral fluids can therefore be infectious during acute CHIKV infections, likely due to hemorrhagic manifestations in the oral/nasal cavities. | Gardner et al., 2015  [8] |
| blood,  saliva,  urine | RT-PCR, ELISA for IgM/IgG | analysis of human saliva for diagnostic | For confirmed CHIK presenting during the 1st week post symptoms onset, CHIKV RNA was detected from 86.1 % (31/36) of blood, 58.3 % (21/36) of saliva and 8.3 % (2/24) of urine. Detection rate of CHIKV RNA was significantly higher in blood compared to saliva. For confirmed CHIK presenting after the 1st week post symptoms onset, CHIKV RNA was detected from 8.3 % (1/12) of blood, 8.3 % (1/12) of saliva and 0 % (0/8) of urine. In contrast to Zika virus (ZIKV), saliva did not increase the detection rate of CHIKV RNA during the 1st week post symptoms onset. In contrast to ZIKV, dengue virus and West Nile virus, urine did not enlarge the window of detection of CHIKV RNA after the 1st week post symptoms onset. Saliva can be used for molecular detection of CHIKV during the 1st week post symptoms onset only if blood is impossible to collect but with a lower sensitivity compared to blood. | Musso et al., 2016  [9] |
| throat swab,  urine | RT-PCR,  IgM,  VI | analysis of throat swab and urine for diagnostic | Detailed protocols for diagnosis of CHIKV for RT-PCR, IgM ELISA, VI, using throat swab and urine specimens. | Raut et al., 2016 [11] |
| blood,  urine | RT-PCR,  ELISA | diagnostic of an acute infection in returning travelers from Cook Islands | CHIK virus was detected in one from two returning travelers in blood and urine by specific RT-PCR. Retrospectively, his history of CHIK fever was confirmed by the presence of the anti-CHIKV IgM and IgG antibodies using the ELISA. | Kondo et al., 2016 [10] |

CHIK = Chikungunya Fever, VI = virus isolation, ELISA = Enzyme Linked Immunosorbent Assay, RT-PCR= Reverse transcription polymerase chain reaction

**Table S2: Analysis of different sampling methods for diagnostic of Dengue virus infection**

| **sample type** | **diagnostic methods** | **reason for analysis** | **results, key findings** | **reference** |
| --- | --- | --- | --- | --- |
| saliva | ELISA,  HAI,  VI | analyse whether saliva could be used for diagnosis of dengue infections | Saliva was collected prospectively from patients presenting with suspected dengue infection 4 to 8 days after the onset of symptoms and assayed for dengue specific IgM and IgG in ELISA. Laboratory diagnosis was based on VI and on HAI and an IgM and IgG ELISA. An overall sensitivity of 92% was obtained for both 1^st^- and 2^nd^-dengue patients (22 of 24), while no patients with non-flavivirus infections (n = 11) and no healthy laboratory donors (n = 17) showed elevation of salivary anti-dengue antibody (100% specificity). Salivary IgG levels correlated well with serum HAI titer (r = 0.78), and salivary IgG levels could be used to distinguish between primary- and secondary-dengue virus infections. | Cuzzubbo et al., 1998 [1] |
| serum,  saliva | IgM, IgG ELISA | analyse whether saliva could be used for diagnosis of recent dengue infections | Recent dengue infection was confirmed in 38 cases. Forty-six serum and saliva specimens were collected from these patients 1-30 days after the onset of symptoms. IgM was detected in 65.8% saliva samples. High rate of positivity (> 80%) was observed for the saliva samples collected > or = 5 days after the onset of the disease. Fifty serum and saliva samples from other 32 patients with rash diseases were also tested and all the specimens were unreactive by ELISA. These results indicate that saliva may be a convenient non-invasive alternative to serum for diagnosis of recent dengue fever infection, especially for epidemiological studies during outbreaks of the disease. | Artimos de Oliveira et al., 1999 [12] |
| plasma,  saliva | RT-PCR IgM, IgG ELISAs,  VI | detection of DENV RNA in an acute Dengue infection with acute bilateral parotitis | Dengue virus RNA was detected by PCR in plasma and saliva and in the supernatant of C6/36 mosquito cells inoculated with either plasma or saliva. For the first time evidence of DENV in the saliva is also provided. | Torres et al. 2000 [13] |
| serum,  saliva | IgM, IgG, IgA ELISAs | evaluate saliva samples as alternative approaches to the serological diagnosis | For the serological diagnosis of DENV infection, the detection of DEN-specific IgM and IgA antibodies in serum and saliva specimens was assessed in 147 patients with symptoms of DEN infection. 72 serum samples were determined to be positive for anti-DEN antibodies by IgM capture ELISA, the routine diagnostic procedure. IgM was detected in the saliva of 65 of the 72 serum IgM-positive cases, 6 of the 75 serum IgM-negative cases, and none of the control group, resulting in a sensitivity of 90.3% and a specificity of 92.0% and demonstrating that salivary IgM is a useful diagnostic marker for DEN infection. Given that saliva is a readily obtainable, noninvasive specimen, detection of DEN-specific salivary IgM should be considered a useful, cheaper diagnostic modality with similar sensitivity and specificity to IgM detection in serum. | Balmaseda et al. 2003 [14] |
| serum,  saliva,  urine | IgM, IgG, IgA ELISAs | analyse the kinetics of IgM, IgA, and IgG in serum, saliva, and urine from adult patients with 1st or 2nd  infection | Similar kinetics were observed in IgM, IgA, and IgG antibodies in saliva and IgA and IgG in urine samples from secondary cases compared with kinetics in serum samples, although the values were lower. No IgG antibody was detected in saliva and urine samples in 1st cases and IgM antibody was not detected in urine samples from either 1st or 2nd infection. All 2nd cases were positive for IgG in saliva and urine samples at day 7. | Vázquez et al., 2007 [15] |
| serum,  saliva | IgM, IgG ELISA | analysis of saliva as non-invasive sample alternative for diagnostic of specific IgM and IgG compared to serum | Salivary IgG antibodies were detected in 93.3% of the serum samples that were positive for anti-dengue IgG antibodies and in none of the serum IgG-negative cases. The detection of IgG in saliva proved to be a promising tool as the sensitivity, specificity, positive predictive value, and negative predictive value were found out to be 93.3%, 100%, 100%, and 83.3%, respectively. Detection of salivary IgM antibodies by ELISA showed 100% sensitivity, 70% specificity, 90.9% positive predictive value, and 100% negative predictive value. | Chakravarti et al., 2007 [16] |
| plasma,  urine | RT-PCR, IgM, IgG ELISA | analysis of acute infection | We successfully detected DENV genome in urine and saliva but not in plasma samples from a Japanese DEN fever patient. The results of the present study suggest that detection of DENV genome in urine and saliva can be an effective diagnostic method, particularly for children with viral haemorrhage. | Mizuno et al., 2007 [18] |
| serum,  saliva | IgM, IgG, IgA ELISAs | analysis of acute- and convalescent infections using filter-paper blood spots | For dengue diagnosis, the highest sensitivity and specificity was  obtained by measuring IgM or IgA in serum or filter-paper blood spots;  intermediate and poor results were obtained in saliva for IgM and IgA  respectively. Detection of IgG alone in serum, filter-paper blood spots, or saliva functioned best for measuring DENV infection.  Detection of IgM and IgA in serum and filter-paper blood spots  yielded optimal results for diagnosis of dengue cases, whereas IgG was the best marker for measuring incidence of DENV infection. | Balmaseda et al., 2008 [17] |
| serum,  saliva,  urine | RT-PCR | analyze the usefulness of urine and saliva samples for early diagnosis | Dengue RNA was detected in serum, urine and saliva samples of  both patients. Patient 1 was infected with DENV-2 and patient 2 with DENV-3. Data presented in this study suggest that urine and saliva could be used as alternative samples for early diagnosis of dengue virus infection when blood samples are difficult to obtain, e.g., in newborns and patients with hemorrhagic syndromes. | Poloni et al., 2010 [19] |
| serum,  saliva | IgA ELISA | analyse the utility of saliva in an assay that detects DENV-specific IgA early in the phase of a dengue infection | Using an antigen capture anti-DENV IgA ELISA technique, we tested saliva samples collected from dengue-confirmed patients. The sensitivity within 3 days from fever onset was over 36% in primary dengue infections. The performance is markedly better in 2nd infections, with 100% sensitivity reported in saliva samples from day 1 after fever onset. Serum and salivary IgA levels showed good correlation. Specificity was found to be 97%.  Our findings suggest that this technique would be very useful in  Dengue endemic regions, where the majority of dengue cases are 2nd. The ELISA is easy to perform, cost effective, and especially useful in laboratories without sophisticated equipment. | Yap et al., 2011 [20] |
| serum,  saliva,  urine | NS1 ELISA,  NS1 strip assay | analyse the utility of saliva and urine for diagnostic of DENV infections | A total of 136 matched serum and urine samples obtained from 55 patients with dengue infection and 30 other febrile illnesses were assayed for DEN NS1 antigen. The urine NS1 ELISA was positive in patients with dengue fever (68.4%) and DEN HF (63.9%), whereas the strip method showed a lower positive rate. The amount of NS1 in urine could possibly reflect protein leak and would be important to monitor over time in patients developing DF or DHF. The discrepancy in positive rate of NS1 in blood between DF and DHF and between 1st and 2nd infection has been reported. We also confirmed our previous report of 98.9% sensitivity of the serum DENV NS1 strip compared to the ELISA method. For urine, the strip method showed only 73.9% to 76.9% sensitivity compared to the ELISA method. Further studies are needed to improve the sensitivity of this method. | Chuansumrit et al., 2011 [21] |
| serum,  urine | RT-PCR,  IgM, IgG ELISA,  VI | analyse the utility of urine samples in the diagnosis of DENV infection by comparing RT-PCR from serially collected urine and serum samples from confirmed DENV cases; compared RT-PCR for urine and serum to IgG and IgM ELISAs for serum and VI from urine | The DENV RNA was detected by RT-PCR in urine and serum of dengue patients. The detection rate of DENV RNA in urine was 25% (2/8) on disease days 0 to 3 and 32% (7/22) on days 4 to 5. The rate was 50% or higher on days 6 to 16, 52% (11/21) on days 6 to 7, 78% (7/9) on days 8 to 9, 80% (4/5) on days 10 to 11, 50% (2/4) on days 12 to 13, and 60% (3/5) on days 14 to 16. The last positive urine sample was on day 16. The detection rates in serum were highest on days 0 to 3 and were greater than 50% on days 0 to 7. Detection rates decreased thereafter, and the last positive detection was on day 11. These results indicate that the time frames for positive detection differ between urine and serum samples, whereby detection rates of 50% or higher are evident between days 6 to 16 for urine samples and days 0 to 7 for serum samples. PCR products were identical between urine and serum samples. The detection of DENV RNA in urine samples by RT-PCR is useful to confirm DENV infection, particularly after viremia disappears. | Hirayama et al., 2012 [24] |
| plasma,  oral swabs,  DBS | RT-PCR,  NS1-ELISA,  IgM, IgG ELISA | analyse the performance of  oral swabs and DBS, compared with plasma, in diagnosing acute dengue and screening for past DENV infection | Oral swabs were less sensitive (IgM: 68.7%, IgG: 91.9%, NS1: 64.7%), but retained good specificity (100%, 92.3%, 95.8%, respectively) compared with plasma. DBS displayed high sensitivity (IgM: 100%, IgG: 96%, NS1: 100%) and specificity (IgM: 75%, IgG: 93%). DENV RNA was amplified from DBS (sensitivity 95.6%) but not from oral swabs. DENV-IgG (indicative of past flavivirus exposure) were detected with moderate sensitivity (61.1%) but poor specificity (50%) in oral swabs from healthy volunteers. | Anders et al., 2012 [23] |
| serum,  saliva,  urine | RT-PCR,  NS1-ELISA,  IgM, IgG ELISA,  VI | analyse the kinetics, and diagnostic potential of DENV-RNA and NS1 in the urine and saliva of dengue patients | DENV-NS1 protein and RNA proved detectable from saliva and urine using tests developed for serum samples. RNA/NS1 detection showed a diagnostic sensitivity of 64%/54% and 60%/56% for urine and saliva, respectively. RNA analyses performed on days 7-13 after onset of symptoms revealed the sensitivity for urine (72%) to be greater than for serum (31%) or saliva (50%). The concentration of urine samples had no impact on RNA detection. Non-invasive sampling enables an alternative approach to dengue diagnostics. The performance of the NS1 antigen assay may be improved by optimizing it for urine and saliva samples. The prolonged excretion of DENV-RNA in urine extends the sampling time window for molecular diagnostics and surveillance. | Korhonen et al., 2014 [25] |
| saliva | ELISA | analyse the diagnostic sensitivity and specificity of the ELISA using saliva | Saliva samples collected from 20 seropositive patients and 20 seronegative patients were subjected to ELISA test for detection of Dengue antibody. A sensitivity of 100% and a specificity of 100% were obtained for making a diagnosis of Dengue infection.  In a developing tropical country like India, such a diagnostic tool has to be encouraged. Further research necessitates the implementation of saliva as a diagnostic tool. | Ravi Banavar, GSV, 2014 [26] |
| serum,  urine | RT-PCR,  IgM ELISA | genomic and serological diagnosis of imported  dengue fever using urine as a non-invasive sample | The results of RT-PCR assay revealed that the serum sample collected on day 5 was DENV-positive; however, the serum sample collected on day 8 and 18 were negative for DENV. The urine sample collected on day 8 and 18 were DENV-positive. We also sequenced the complete DENV genome (10723 bp) from the urine sample. We provided the first evidence of a DENV-2 infection that was imported from India to a non-endemic city of China, investigated the DENV genome detection having a longer timeframe for positive detection in urine sample compared to previous studies. | Ma et al., 2014 [27] |
| serum,  urine | RT-PCR | analyse two RNA extraction procedures from urine and investigated the stability of DENV RNA in urine and serum up to 1 year at different storage temperatures | DENV RNA in both serum and urine was stable at room temperature up to 1 month and at 4 °C and -20 °C for at least 1 year. The detection rate by RT-PCR on urine was 50 % (4/8) until day 7, 100 % (6/6) between 1 and 3 weeks after symptom onset, and 25 % (2/8) thereafter. Generally, DENV RNA concentrations are higher in serum than in urine up till day 7, switching to lower concentrations in serum thereafter. Peak concentrations in urine are reached around day 10, and RNA becomes undetectable 3 to 4 weeks following disease onset. This diagnostic tool is of added value in clinical settings by extending the period during which DENV infections are diagnosed by RT-PCR. | Van den Bossche et al., 2015 [28] |
| saliva | IgG | analysis of a stacking flow platform for detection of specific IgG | We developed a stacking flow platform for single-step detection of a target antibody in salivary fluid. Stacking flow circumvents the need for separate sample pre-treatments, such as filtration or centrifugation, which are often required prior to testing saliva samples using paper-based immunoassays. We have successfully applied the stacking flow device to detect dengue-specific immunoglobulins that are present in salivary fluid. | Zhang et al., 2015 [29] |
| serum,  urine | RT-PCR,  IgA RDT,  IgM, IgG ELISA | analyse the utility of NS1 ELISA for diagnostic of DENV infection for urine samples | NS1 antigen was detected by ELISA in the urine samples obtained from patients after 2-17 days of disease onset. Positive detection rates of NS1 antigen ranged between 13-43%. Based on real-time RT-PCR, positive detection rates of viral genome in the urine samples ranged between 20-33% on days 0 to ≥15. On days 11 to ≥15 after the disease onset, NS1 antigen was detected at similar rates in serum and urine samples. Additionally, NS1 antigen was detected in 2 urine samples, but not in the serum samples, on days 7 and 16 after the onset of the disease. The results confirm the applicability of NS1 antigen detection in urine samples using ELISA to diagnose acute DENV infection and suggests that the assay is potentially useful when only limited amounts of serum samples are available and in limited resource settings. | Saito et al., 2015 [22] |
| plasma,  saliva,  urine | RT-PCR,  NS1 ELISA,  IgM, IgG, IgA ELISAs,  VI | analyse the profiles of the anti-DENV IgG, IgM and IgA, the NS1 antigen, DENV RNA and of the infectious virus in plasma, saliva and urine in children | Quantitative RT-PCR, NS1 antigen capture and ELISA serology for anti-DENV antibody (IgG, IgM and IgA) detection were performed in parallel on the three body fluids. RT-PCR and NS1 tests demonstrated an overall sensitivity of 85.4%/63.4%, 41.6%/14.5% and 39%/28.3%, in plasma, urine and saliva specimens, respectively. When urine and saliva samples were collected at the same time-points and tested concurrently, the diagnostic sensitivity of RNA and NS1 detection assays was 69.1% and 34.4%, respectively. IgG/IgA detection assays had an overall sensitivity of 54.4%/37.4%, 38.5%/26.8% and 52.9%/ 28.6% in plasma, urine and saliva specimens, respectively. IgM were detected in 38.1% and 36% of the plasma and saliva samples but never in urine. Although the performances of the different diagnostic methods were not as good in saliva and urine as in plasma specimens, the results obtained by RT-PCR and by anti-DENV antibody ELISA could well justify the use of these two body fluids to detect dengue infection in situations when the collection of blood specimens is not possible. | Andries et al., 2015 [30] |
| plasma,  saliva,  urine | ELISA,  NS1-ELISA,  RDT | analyse the RDTs for the detection of NS1 antigen and anti-dengue IgG, IgM and IgA in urine and saliva | The RDTs demonstrated an overall sensitivity of 15.5 %/27.9 %/10.7 % for NS1/IgG/IgA detection in urine samples and 20.4 %/4.8 %/11 %/  6.2 % for NS1/IgG/IgM/IgA detection in saliva samples. Compared to the in-house NS1 ELISA, the results obtained with the NS1 RDT demonstrated a good correlation with urine samples but not with saliva specimens. RDTs designed for antibody detection in saliva and urine were extremely specific (100 %), but less sensitive than the in-house ELISAs (i.e., reduction of the overall sensitivity by 12.2 % for the RDT designed for IgG detection in urine and by 23.7 % for the RDT detecting anti-DENV IgM in saliva). IgM were not detected in urine, either by RDT or ELISA. This study suggests that these commercial RDT kits would require further improvement to increase the sensitivity. | Andries et al., 2016 [31] |
| serum,  urine | RT-PCR,  IgA ELISA | analyse the DENV-IgA antibody profiles in urine samples from DF and SD patients, and to clarify the potential relationship between IgA and the severity of disease | In this study, 262 serial urine samples from 78 laboratory-confirmed patients were assayed by a commercial IgA kit against DENV. The total positive rate of IgA in urine was 59%. DENV-specific IgA was detected in urine from day 2 to day 13 after the onset of illness in DF patients.  While for SD patients, anti-DENV IgA could be detected till day 14. The positive rate of IgA in patients with 2nd infection was higher than that in patients with 1st infection. During 4-7 days after the onset of illness, the IgA positive rate of SD patients was significantly higher than that of DF patients. Especially, the intensity of IgA signal in SD patients was obviously stronger than that in DF patient at the recovery stage. DENV-specific IgA antibodies in urine might be a warning sign for SD. | Zhao et al., 2016 [32] |

DENV= Dengue virus, VI = virus isolation; HAI = hemagglutination inhibition (HAI) assay, DBS = dried blood spots, RDT = rapid diagnostic test, DF = dengue fever, SD = severe dengue, HF = haemorrhagic fever, ELISA = enzyme-linked immunosorbent assay, RT-PCR= Reverse transcription polymerase chain reaction,

**Table S3: Analysis of different sampling methods for diagnostic of Ebola/VHF virus infections**

| **sample type** | **diagnosticmethods** | **reason for analysis** | **results, key findings** | **reference** |
| --- | --- | --- | --- | --- |
| saliva,  serum | ELISA,  PRNT,  VI | analysis of formalin inactivated **Rift Valley Fever Virus** vaccine efficacy in sheep | Challenge control sheep developed clinical disease and detectable RVF viremia after exposure. Virus was isolated from saliva of 1 challenge control sheep and virus was transmitted by contact exposure to 1 of 4 seronegative contact-control sheep. | Harrington et al., 1980 [33] |
| lung,  kidney, salivary  gland,  liver,  urine,  saliva,  feces | IFA,  AG test | **Hantaan virus** pathogenesis studies in rodents | *Apodemus agrarius* rodents inoculated with Hantaan virus by the intramuscular route experienced viremia for about 1 week beginning on day 7. After 3 weeks, immunofluorescent and neutralizing antibodies were present and no mouse ever developed signs of acute illness. Virus was recovered from lung, kidney, salivary gland, and liver, and virus excretion in urine, saliva, and feces occurred from about day 10 through day 360 (urine) post-inoculation. Antigen, but not infectious virus, was persistent in lung tissue for as long as 1 year. | Lee et al., 1981 [34] |
| urine,  throat swaps,  blood | VI,  IFA | diagnostic of an acute **Lassa virus** infection imported into UK | Lassa virus was not detected in urine during the first 3 weeks but then appeared and reached a peak during the 6th week, with continuing excretion for 67 days after the onset of illness. VI and serological studies were made on specimens of blood, urine, and throat washings collected during the acute and convalescent phases of the illness.  The highest level of virus in blood (10^5.2^ median TCID/ml) was found in the sample taken 11 d.p.o.; the level then declined,and the virus was undetectable by day 32. The peak level of virus (10^4^ median TCID/ml) was found in the urine on day 36, but levels ranging from 10^2^ to 10^4^ median tissue culture infective dose/ml were evident from days 22 to 53. Throat washings taken throughout the patient's isolation did not yield any virus. | Emond et al., 1982 [35] |
| urine,  blood,  oral swaps,  brain,  spleen, kidneys, salivary glands | IFA,  AG test | **Junin virus** pathogenesis studies in rodents | 85 *C. musculinus* were inoculated intranasally at birth with 100 TCID50 Junin virus and observed for 480 days. Infected animals showed an increased mortality rate of up to 70% between days 24-40 post-infection. From day 14 post-infection until day 480, Junin virus was recovered from blood, urine, and oral swab in all animals checked at any time. By day 480 post-infection, 100% of survivors showed wides-pread viral dissemination in brain, spleen, kidneys, and salivary glands. There was marked reduction in reproductive efficiency among infected animals. | Vitullo et al., 1987 [37] |
| urine,  saliva | IFA,  AG test,  VI | **Junin virus** pathogenesis studies in rodents | Intranasal infection in its adult natural host, *Calomys musculinus* (90-120 days) by Junin virus did not produce mortality or illness during the 150-day period of observation. From day 21 to 150 after infection, 50% of the animals showed viral persistence with shedding of virus in both urine and saliva. The remaining half became seropositive, and no infectious virus was recovered from them. | Vitullo et al., 1990 [38] |
| urine | IFA,  AG test | diagnostic of acute cases of **Hanta virus** patients with HFRS | Hanta virus antigen was detectable in the urine sediment epithelium in 7 of the 8 patients from the seventh to the 32 day of the follow-up. Fluorescent bright-emerald dots could be seen in the urine sediment cells, situated on the cell surface, in the cytoplasm round the nucleus and in the periphery. The efficacy of the method was 87.5 +/- 12.5%. This method permits monitoring the virus persistence and its isolation from the patient during the acute period of the disease and early convalescence period. | Vereta LA, Elisova, 1990 [40] |
| urine,  serum | PCR,  VI,  IFA | diagnostic of an acute **Lassa virus** infection imported into Germany | In all 5 serum specimens and 4 of five urine specimens of patients with acute Lassa fever, viral RNA could be demonstrated. Our data suggest that PCR may be applied as an alternative to virus isolation in the rapid diagnosis of Lassa fever. | Lunkenheimer et al., 1990 [36] |
| urine | animal model | **Marburg virus** infection studies in guinea pigs and M. rhesus monkeys | Experiments in guinea pigs and M. rhesus monkeys showed the possibility of contact infection of animals with Marburg virus. 2^nd^ infection occurred most intensively when the monkeys were kept together but was also shown to be possible when the animals were separated but placed in the direction of the air flow from the sick monkeys as well as by "nose-to-nose" contact excluding the alimentary mode of transmission and the role of the agent excreted in the urine. | Pokhodiaev et al., 1991 [48] |
| urine,  blood | IFA,  AG test | pathogenesis study of acute cases of **Hanta virus** patients with HFRS | 135 urine samples from 50 patients with HFRS were examined at different periods of the disease. Antibodies were demonstrable in all urine specimens from HFRS patients for 13 days. In 14-20 days they could be detected in half of the patients, and no antibodies could be demonstrated since the 21^st^ day on. The results of urine examination from healthy subjects and some patients with other clinical diagnoses were negative same as controls with normal antigen. The dynamics of antibody titres in the patients' urine differed from that in the blood and was considered as "decreasing" similarly as the clinical disease. The antibody excretion in the urine coincided with the period of renal structure damage and stopped when the normal renal function was restored. | Vereta et al., 1993 [41] |
| urine,  brain tissue | IFA,  VI,  PCR | diagnostic of an fatal **Hantaa virus** infection in Slovenia | Recently, a hantavirus was isolated from the urine and brain tissue of a fatal case of HFRS. Furthermore, the specificity of the isolates was confirmed by PCR. This appears to be the first isolation of a strain of prototype Hantaan virus from a fatal case of HFRS in Europe. | Avsic-Zupanc et al., 1994 [42] |
| blood,  saliva | IgG IFA,  AG ELISA | prevalence of **Junin virus** in rodent populations in the epidemic areas | Approximately half of infected rodents simultaneously carried serum antibody and antigen in blood and saliva, some for 29-61 days. These observations suggest that most infections were acquired through horizontal transmission and that aggressive encounters among adult, male *C. musculinus* in relatively densely populated roadside and fence-line habitats are an important mechanism of transmission of Junin virus within reservoir populations. | Mills et al., 1994 [39] |
| oropharyngeal swaps,  urine |  | **Guanarito virus** infection studies in rodents | 39 laboratory-reared cane mice each were inoculated subcutaneously with 3.0 log10 PFU of the Guanarito virus. No lethality was associated with infection in any animal, regardless of age at inoculation. The 13 newborn, 14 weanling, and 8 of the 12 adult animals developed chronic viremic infections characterized by persistent shedding of infectious virus in oropharyngeal secretions and urine. These findings  indicate that Guanarito virus infection in *Z. brevicauda* can be chronic and thus support the concept that this rodent species is the natural reservoir of Guanarito virus. | Fulhorst et al., 1999 [136] |
| saliva,  urine,  feces,  blood | RT-PCR | **Marburg virus** infection studies in guinea pigs | Marburg virus in guinea pig saliva, urine, and feces showed that as early as by the end of incubation period the virus concentrations in the feces and saliva were 2.3-3.3 lg LD50. In the blood the content of the virus was high and increased by the end of the disease, while the concentrations in the urine, saliva, and feces were virtually the same throughout the disease. | Chupurnova et al., 2000 [49] |
| blood,  urine,  heart,  liver,  lung,  spleen, kidney,  stool | VI,  AG assay,  IgG assay  PCR | significance of **Hanta virus** infection in domestic pigs | Hanta virus antigen and antibody were detected in the heart, liver, lung, spleen, kidney, blood, urine, and stool of domestic pigs as well as in the sewage of the pigpen facilities. . The positivity rate of HFRSV antigen ranged from 3.3% to 5.0% in the organ of pigs, and HFRSV positivity rate in the blood, urine, stool, and sewage was 3.7%, 7.0%, 2.5%, and 5.6%, respectively, with a total serum antibody positivity rate of 2.0%. Domestic pigs can be the host of HFRSV for the viral transmission. | Yang et al., 2004 [43] |
| saliva,  serum | RT-PCR,  IFA, | analysis of oral fluid for **Ebola virus** during an outbreak in the Republic of Congo | We failed to detect antibodies against Ebola in the oral fluid specimens obtained from patients whose serum samples were seropositive. All patients with positive serum RT-PCR results also had positive results for their oral fluid specimens. This study demonstrates the usefulness of oral fluid samples for the investigation of Ebola outbreaks, but further development in antibodies and antigen detection in oral fluid specimens is needed before these samples are used for filovirus surveillance activities in Africa. | Formenty et al., 2006 [52] |
| nasopharyngeal aspirates, blood,  urine,  lung | RT-PCR,  hybridizion arrays | evaluation of a chip based hybridization array for analysis of acute cases for multiple pathogens incl. **VHFs** | Analysis of nasopharyngeal aspirates, blood, urine, and tissue from persons with various infectious diseases confirmed the presence of viruses and bacteria identified by other methods, and implicated Plasmodium falciparum in an unexplained fatal case of hemorrhagic feverlike disease during the Marburg hemorrhagic fever outbreak in Angola in 2004-2005. | Palacios et al., 2007 [63] |
| saliva,  stool,  semen, breast milk, tears,  nasal blood, and skin swab | RT-PCR,  IgG ELISA,  VI | analysis of various clinical specimens from 26 laboratory-confirmed cases of Ebola HF, for the presence of **Ebola virus** | Virus was detected by culture and/or RT-PCR in 16 of 54 clinical specimens (including saliva, stool, semen, breast milk, tears, nasal blood, and a skin swab) and in 2 of 33 environmental specimens. We conclude that EBOV is shed in a wide variety of bodily fluids during the acute period of illness but that the risk of transmission from fomites in an isolation ward and from convalescent patients is low when currently recommended infection control guidelines for the viral HFs are followed. | Bausch et al., 2007 [53] |
| saliva,  serum | RT-PCR,  IFA,  sequenc-ing | analysis of saliva from HFRS patients for **Hantavirus** | We collected saliva and plasma from 14 hospitalized NE patients with verified Puumala virus (PUUV) infection. PUUV RNA was detected in saliva from 10 patients (range 1,530-121,323 PUUV RNA copies/ml) by quantitative RT-PCR. The PUUV S-segment sequences from saliva and plasma of the same patients were identical. Our data show that hantavirus RNA could be detected in human saliva several days after onset of disease symptoms and raise the question whether interhuman transmission of hantavirus may occur through saliva. | Pettersson et al., 2008 [44] |
| saliva,  urine | RT-PCR | diagnostic of an acute **CCHF virus** infection | The genome of CCHF virus was detected in the saliva from 5 of the 6 patients and in the urine from two of the 3 patients. The levels of viral load in the saliva and urine samples were similar to those in the blood samples in all but one patient, in whom higher levels were detected in blood compared to saliva or urine. This study shows that during human infection with CCHF virus, viral genomes are present in the saliva and urine. | Bodur et al., 2010 [61] |
| saliva, plasma, serum | IgA IFA,  RT-PCR,  IH | analysis of salivary IgA in relation to viral antigen in the saliva by testing **Hantavirus** specific IgA, and RNA in saliva in acute HFRS patients | In saliva samples, PUUV specific IgA was detected in 12 of 33 (36%) patients with HFRS and 20 (61%) were PUUV RNA positive. There was a statistically significant inverse association between the presence of salivary IgA antibodies and PUUV RNA in the saliva. PUUV-specific IgA in saliva was not found in a long-term follow-up, while PUUV IgA in serum was detected in 3 patients, 28-32 months after the initial study.  Notably, both PUUV RNA and PUUV nucleocapsid antigen were detected in endothelial cells within the parotid gland of a diseased patient with HFRS. | Pettersson et al., 2011 [45] |
| blood,  throat swab, urine,  feces | RT-PCR | diagnostic of an acute novel **Bunya virus** infection in China | HF-like illness caused by a novel Bunyavirus, Huaiyangshan virus (HYSV, also known as Severe Fever with Thrombocytopenia virus [SFTSV] and Fever, Thrombocytopenia and Leukopenia Syndrome [FTLS]), has recently been described in China. Viral RNA was also detectable in throat, urine, and fecal specimens of a substantial proportion of patients, including all fatal cases assayed. | Zhang et al., 2013 [65] |
| liver,  spleen,  lung,  kidney,  colon,  feces | RT-PCR | anlysis of bats for **Marburg virus** after infection of a tourist visiting the cave in Uganda | Between Aug. 2008 and Nov. 2009, 1,622 bats (*Rousettus aegyptiacus*) were captured and tested for Marburg virus. Q-RT-PCR analysis of bat liver/spleen tissues indicated, 2.5% of the bats were actively infected, seven of which yielded Marburg virus isolates. Moreover, Q-RT-PCR-positive lung, kidney, colon and reproductive tissues were found, consistent with potential for oral, urine, fecal or sexual transmission. | Amman et al., 2012 [50] |
| urine,  serum | RT-PCR,  IFA,  IgM, IgG ELISAs | review of **CCHFV** infections from Kosova in 2008 – 2009 regarding viremia in urine | CCHF virus was detected in urine samples, and these patients were found to have prolonged viremia. The detection of CCHF in urine, as well as the prolonged viremias seen, are important for clinicians to know, as they may have public health implications with regard to the risk of infection, as well as provide insights into the biology and pathophysiology of infection. Further studies are required regarding the pathogenesis of this virus. | Thomas et al., 2012 [62] |
| mucosal swab,  urine,  feces | RT-PCR,  IgG ELISA,  IH | analysis of infectivity of **Marburg virus** in fruit bats | Egyptian fruit bats (*Rousettus aegyptiacus*) were inoculated sub-cutaneously (n = 22) with Marburg virus (MARV). The virus was detected in 15 different tissues and plasma but only sporadically in mucosal swab samples, urine, and fecal samples. Neither sero-conversion nor viremia could be demonstrated in any of the in-contact susceptible bats (n = 14) up to 42 days after exposure to infected bats. | Paweska et al., 2015 [51] |
| plasma,  blood,  urine,  sweat,  saliva, conjunctival swabs,  stool | RT-PCR,  IgM, IgG IFA,  VI | montoring of a recovered patient after **Ebola virus** infection | The discharge of an convalescence Ebola patient was delayed owing to the detection of viral RNA in urine (day 30) and sweat (at the last assessment on day 40) by means of PCR assay, but the last positive culture was identified in plasma on day 14 and in urine on day 26. | Kreuels et al., 2014 [54] |
| serum,  saliva | IFA | analyse the seroprevalence of **Hantavirus** antibodies in known risk group in the UK | Prevalence of antibodies against hantaviruses in serum and saliva of adults living or working on farms in Yorkshire, UK. Of a total 119 individuals tested, 9 (7.6%) were seropositive for hantavirus antibodies. | Jameson et al., 2014 [46] |
| blood,  urine | RT-PCR | analyse the **Ebola virus** load in a convalescenct patient | Ebola viral load started to steadily decrease in the blood after treatment and became undetectable by day 19 after admission, while it persisted longer in urine samples. | Petrosillo et al., 2015 [55] |
| urine | RT-PCR | evaluation of a bead array assays for detection of multiple bat-borne viruses incl. **Ebola virus** and **Nipha** | These assays detect up to 11 viral RNA's simultaneously in urine samples collected from wild bat populations in Australia and Bangladesh. | Boyd et al., 2015 [64] |
| whole-blood, plasma,  urine | RT-PCR,  film-array | evaluation of a film-array assays for detection of **Ebola virus** | Rates of agreement between FilmArray and qRT-PCR results for clinical specimens from patients with EVD were 85% (23/27 specimens) for whole-blood specimens, 90% (18/20 specimens) for whole-blood specimens tested by FilmArray testing and matched plasma specimens tested by qRT-PCR testing, and 85% (11/13 specimens) for urine specimens. Among 60 specimens, 8 discordant results were noted, with EBOV nucleic acids being detected only by FilmArray testing in four specimens and only by qRT-PCR testing in the remaining four specimens. | Southern et al., 2015 [59] |
| blood,  saliva,  urine,  faeces | RT-PCT,  IFA | analysis of **Hantavirus** persistence in wild rodents | In a monthly capture–mark–recapture study, we analysed 18 bank voles for the presence and relative quantity of PUUV RNA in the excreta and blood from 2 months before up to 8 months after seroconversion. The proportion of animals shedding PUUV RNA in saliva, urine and faeces peaked during the first month after seroconversion, but continued throughout the study period with only a slight decline. The quantity of shed PUUV in reverse transcription quantitative PCR (RT-qPCR) positive excreta was constant over time. In blood, PUUV RNA was present for up to 7 months but both the probability of viraemia and the virus load declined with time. Our findings contradict the current view of a decline in virus shedding after the acute phase and a short viraemic period in hantavirus infection – an assumption widely adopted in current epidemiological models. | Voutilainen et al., 2015 [47] |
| blood,  saliva,  urine, aqueous humor, semen, breast milk,  stool,  vaginal fluid | RT-PCR,  IgM, IgG IFA,  VI | review the evidence on modes of **Ebola virus** shedding and transmission | Ebola virus has been isolated by cell culture from blood, saliva,  urine, aqueous humor, semen, and breast milk from infected or convalescent patients. Ebola virus RNA has been noted in the following body fluids days or months after onset of illness: saliva (22 days), conjunctiva/tears (28 days), stool (29 days), vaginal fluid (33 days), sweat (44 days), urine (64 days), amniotic fluid (38 days), aqueous humor (101 days), cerebrospinal fluid (9 months), breast milk (16 months [preliminary data]), and semen (18 months). | Vetter et al., 2016 [56] |
| blood,  semen,  sweat,  urine, conjunctiva swap,  vagina swap | RT-PCR | analysis of different body fluid for presence of **Ebola virus** genome for estimating the infection risk to health-care providers | We tested a total of 555 specimens: 103 from the axilla, 93 from blood, 92 from conjunctiva, 54 from forehead, 105 from mouth, 17 from the rectum, one from semen, 69 from urine, and 21 from the vagina. The median time from Ebola treatment unit discharge to  specimen collection was 142 days. 15 participants had a total of 74 swabs taken less than 100 days from discharge. The semen sample from one participant tested positive for Ebola virus at 114 days after discharge from the treatment unit; specimens taken from the axilla, blood, conjunctiva, forehead, mouth, rectum, and urine of the same participant tested negative. All specimens from the other 111 participants tested negative. | Green et al., 2016 [58] |
| plasma,  urine | RT-PCR | analysing the stability of **Ebola virus** RNA in human blood and urine under environmental conditions | Our results indicate that EBOV RNA is stable in EDTA plasma samples collected and tested in the environmental conditions of West Africa. In the early phase of Ebola disease, blood sampling is probably more sensitive and reliable than oral swabbing and should be used whenever possible. Plasma samples can be tested for up to 18 days after collection, even if stored at ambient temperature, and positive results can still be reported. In the 13 urine samples, initial Ct values had a range of 18.2-35.5 (mean 28.6, 95% CI 25.7-31.5). RNA in urine was undetectable by days 10 and 14 for initial Ct counts of 19.0 and 18.0, respectively. For equivalent Ct values for blood, RNA was detectable for at least 18 days. | Janvier et al., 2016 [60] |
| blood,  urine, aqueous humor, semen, breast milk,  feces,  vaginal fluid | RT-PCR,  AG-ELISA, IFA,  VI | review the persistence of **Ebola virus** in various body fluids during convalescence | EBOV has been isolated from semen, aqueous humor, urine and breast milk 82, 63, 26 and 15 days after onset of illness, respectively. Viral RNA has been detectable in semen (day 272), aqueous humor (day 63), sweat (day 40), urine (day 30), vaginal secretions (day 33), conjunctival fluid (day 22), faeces (day 19) and breast milk (day 17). | Chughtai et al., 2016 [57] |

d.p.o = days post onset of disease, TCID = tissue culture infective dose, AG-Test = antigen capture test, HFRS = hemorrhagic fever with renal syndrome, PFU = plaque-forming units; CDC = Centers for Disease Control and Prevention; HF = Hemorrhagic fever, IH = immune histochemistry, IFA = immune fluorescence assay, AG-ELISA = antigen ELISA, WB = Western blot, RVF = Rift Valley Fever, PRNT = plaque reduction neutralisation test; CCHF = Crimean Congo hemorraghic fever

**Table S4: Analysis of different sampling methods for diagnostic of West Nile virus infection**

| **sample type** | **diagnostic methods** | **reason for analysis** | **results, key findings** | **reference** |
| --- | --- | --- | --- | --- |
| urine | RT-PCR,  VI,  NT | research on pathogenesis in hamsters | The chronically infected rodents shed virus in their urine for up to 8 months, despite the disappearance of viremia and the development of high levels of neutralizing antibodies. Complete sequence analyses were done to compare four WNV isolates from the urines of persistently infected hamsters with the wild-type parent virus (NY 385-99). Nucleotide changes, ranging from 0.05% to 0.09%. The genetic changes associated with renal tropism were also accompanied by a loss of virulence for hamsters and a change in plaque morphology. | Ding et al., 2005 [66] |
| urine,  kidney tissue | VI,  IH | research on pathogenesis in hamsters | Hamsters experimentally infected with WNV developed chronic renal infection and persistent shedding of virus in urine for up to 8 months, despite initial rapid clearance of virus from blood and the timely appearance of high levels of specific neutralizing antibodies. Infectious WNV could be recovered by direct culture of their urine and by co-cultivation of kidney tissue for up to 247 days after initial infection. Comparison of WNV isolates from serial urine samples from individual hamsters over several months indicated that the virus underwent both genetic and phenotypic changes during persistent infection. | Tesh et al., 2005 [67] |
| urine,  kidney tissue | VI,  IH | research on pathogenesis in hamsters | Infectious WNV could be cultured from their urine for up to 52 days. Immunohistochemical examination of kidneys of viruric animals showed foci of WNV antigen in renal tubular epithelial and vascular endothelial cells. These findings are compatible with virus replication and persistent infection of renal epithelial cells | Tonry et al., 2005 A [68] |
| urine,  CHF,  serum | RT-PCR,  IgM, IgG, ELISA  VI | diagnostic of acute infection | We report West Nile virus (WNV) RNA in urine collected from a patient with encephalitis 8 days after symptom onset. Viral RNA was detected by RT-PCR. Sequence and phylogenetic analysis confirmed the PCR product to have > or = 99% similarity to the WNV strain NY 2000-crow3356. | Tonry et al., 2005 B [75] |
| urine,  saliva,  feces | VI,  ELISA,  IH | research on pathogenesis in eastern chipmunks | WNV also was detected in urine, saliva, and feces of some chipmunks. These data suggest chipmunks might play a role in enzootic WNV cycles and be an amplifying host for mosquitoes that could infect humans. | Platt et al., 2007 [71] |
| oral cavities,  rectal cavities,  urine | VI | research on pathogenesis in fox squirrels | WNV was isolated from the oral and rectal cavities of all squirrels and from urine that was opportunistically collected from 5 squirrels. The largest quantity of WNV recovered from swabs of the oral cavity and urine was 10(3.1) PFU. The longest periods after exposure that WNV was isolated from the oral cavity and urine from a squirrel were 22 and 17 days p.e., respectively. WNV RNA was also detected in kidney tissue in 1 squirrel 29 days p.e., suggesting that fox squirrels can be persistently infected. Collectively these observations provide further evidence that squirrels can contribute to the natural history and epidemiology of WNV, especially in peridomestic environments. | Platt et al., 2008 [70] |
| urine | RT-PCR,  VI | research on pathogenesis in hamsters | Hamsters experimentally infected with the neuroinvasive WNV strain NY385-99 frequently develop persistent renal infection and viruria. Viruses recovered from the urine of such animals no longer cause neurological disease when inoculated into naïve hamsters. Our findings indicate that WNV underwent additional genetic changes during serial passage in hamsters, but there was no reversion to neurotropism and virulence. | Wu et al., 2008 [74] |
| urine,  oral swaps,  salivary glands,  kidney tissue | RT-PCR,  NT | research on pathogenesis in fox squirrels model | This virus was also isolated from swabs of the oral and rectal cavities, and urine swabs between day 5 and 9 postexposure (p.e.) in amounts as high as 10(2.0), 10(2.8), and 10(2) PFU, respectively. WNV RNA was detected in salivary gland and/or kidney tissue of three squirrels between day 65 and 72 p.e. in the presence of WNV neutralizing antibody, suggesting that long-term persistent infection occurs in fox squirrels. These observations justify further studies to determine if nonarthropod transmission and long-term persistent infection occur naturally in fox squirrels and contribute to trans-seasonal maintenance of WNV. | Tiawsirisup et al., 2010 [73] |
| urine | RT-PCR | surveillance study within years after infection | West Nile virus (WNV) RNA was demonstrated in 5 (20%) of 25 urine samples collected from convalescent patients 573-2452 days (1.6-6.7 years) after WNV infection. Four of the 5 amplicons sequenced showed >99% homology to the WNV NY99 strain. These findings show that individuals with chronic symptoms after WNV infection may have persistent renal infection over several years. | Murray et al., 2010 [76] |
| urine | RT-PCR | diagnostic of acute neurological infections | According to the national case definition, six cases with acute neurological disease were confirmed in hospitalized patients, and four of them died; one of these was only 34 years old. In two cases, WNV RNA was detected in urine, suggesting renal involvement. Sequence analysis showed lineage 1 and 2 circulation. | Magurano et al., 2012 [80] |
| urine | RT-PCR,  TMA | surveillance study within 5 month after infection | We detected WNV RNA in urine samples from 1 of 63 persons (1.6%) tested <5 months after initial acute WNV infection. Our results indicate infrequent presence of WNV RNA in urine among persons within months after acute WNV infection. | Baty et al., 2012 [78] |
| urine | RT-PCR,  TMA | surveillance study after 6 years of infection | WNV RNA was not detected in urine in any of our 40 cohort members tested 6 years following initial WNV disease. Further studies are needed to determine if and for how long WNV infection persists in humans and to evaluate the clinical relevance therein. | Gibney et al., 2011 [79] |
| urine | VI | research on pathogenesis in mouse | Mice were infected with WNV strain H8912, previously cultured from the urine of a persistently infected hamster, to determine its patho-genesis in a murine host. WNV is highly attenuated in both neuro-invasiveness and neurovirulence in mice. It induces a low and delayed anti-viral response in mice and preferentially persists in the kidneys. | Saxena et al., 2013 [72] |
| urine,  plasma,  CSF | RT-PCR,  IgM, IgG ELISAs, PRNT,  NAT | analysis of the RT-PCR for routine testing during outbreak in 2012 | In 4 of 17 patients with symptomatic infection for whom follow-up laboratory investigation was done, WNV RNA was still detectable in urine 23–31 days after symptom onset, while in blood donors, WNV RNA was detectable in urine up to 10 days after a WNV NAT–positive blood donation. Overall, the WNV RNA load in urine was higher than in plasma. In fact, in WNV RNA–positive urine samples, the half of patients with symptomatic WNV infection, defined as patients with WN neuroinvasive disease or West Nile fever, had median WNV RNA load was 30 000 GE/mL (range, 250–2 500 000 GE/mL), while the median WNV RNA load in WNV RNA–positive plasma samples was 2000 GE/mL (range, 60–60 000 GE/mL); mean WNV RNA loads (±SD) were 200 000 ± 530 000 GE/mL in urine and 7500 ± 14 600 GE/mL in plasma No relationship was observed between the time from symptom onset and the WNV RNA load in urine and in plasma. The WNV RNA load in urine was higher in patients with WN neuroinvasive disease than in those with West Nile fever, while patients with WN fever had longer excretion of WNV RNA in urine than patients with WN neuroinvasive disease (up to 14 days and up to 31 days, respectively, after symptom onset), although these differences were not statistically significant. | Barzon et al., 2013 [77] |
| urine | RT-PCR,  VI | review | Summarize and discuss the results of studies in the literature that investigated WNV infection of the kidney in humans and in animal models and WNV excretion with urine, the potential damage to the kidney caused by WNV infection, the risk of WNV disease in kidney transplant recipients, the significance of detecting WNV in urine and its use in the diagnosis of WNV infection, and kidney involvement by other mosquito-borne flaviviruses. In patients with acute WNV infection, WNV is generally detectable in urine at a higher load  (10^2^ -10^7^ RNA copies/ml) and for a longer time (up to 1 month after symptom onset) than in plasma (50 - 5 x 10^5^ RNA copies/ml). | Barzon et al., 2013 [82] |
| urine | RT-PCR,  VI | surveillance study | Out of the 17 WNV RNA-positive urine specimens that were  analysed in this study WNV was isolated in 6 cases, including 2 with WNV lineage 1 infection and 4 with WNV lineage 2, and of these 6 patients, 3 had WN-ND, 1 had WN-F, and 2 were asymptomatic blood donors, thus demonstrating that infectivity of WNV in urine was not restricted to viral lineages or strains and was independent of the severity of disease. In all 6 cases, WNV was isolated from urine in Vero E6 cells and in BHK21 cells with equal efficiency. | Barzon et al., 2014 [81] |
| urine | RT-PCR,  VI | diagnostic of acute infections | WNV RNA was detected in 40% of the samples from 35 patients with acute WNV infection with cycle threshold (CT) values ranging from 27.0 to 39.9. WNV was isolated from 2 of 4 urine samples with low CT (<30). Viral load was not associated with patients' age, sex, day of illness, presence of WNV antibodies, and neurological symptoms. | Papa et al., 2014 [86] |
| plasma,  urine | nested PCR,  RT-PCR,  IF,  PRNT | patients with acute kidney injury (AKI) and chronic kidney disease | WNV RNA and specific immunoglobulins were detected in 7 (5.7%) and 5 (4.1%) individuals, respectively. The AKI patients with WNV RNA in blood and urine had underlying diseases requiring immunosuppressive therapy and demonstrated moderate to high viral loads. | Ergunay et al., 2015 [84] |
| urine,  blood,  CSF | RT-PCR,  ELISA,  NS1-ELISA,  VI,  IF,  NT,  others | review the latest developments and challenges in the  diagnosis | The direct diagnosis of acute WNV infection is based on the detection of WNV RNA by nucleic acid amplification methods. WNV RNA may be detected in peripheral blood from 2–3 days to 14–18 days post infection, although in approximately 70–80% of cases of infection, viral RNA is no longer detectable in blood at the time of symptom onset. Testing urine samples is more sensitive, since WNV RNA is detectable in 40–50% of patients with WN fever or WN neuroinvasive disease. Molecular methods include highly sensitive NATs for the screening of blood and organ donors and RT-PCR or pan-flavivirus RT-PCR assay for the diagnosis in patients with symptoms. Low viral load at the time of symptom onset and the high diversity of WNV represent the major hurdles for molecular testing. WNV can be isolated from the serum samples of WNV-infected patients within the first 2–3 days post infection, before the appearance of neutralizing antibodies. The virus can be isolated also from the urine samples independently from the presence of antibodies in serum. | Barzon et al., 2015 [83] |
| serum,  urine | RT-PCR | diagnostic of acute infection | The laboratory serologically confirmed 11 acute human infections during the 2014 seasonal period. In three patients with neurological symptoms, viral RNA was detected from both urine and serum specimens, albeit for a longer period and in higher copy numbers with urine. Phylogenetic analysis of the NS3 genomic region of three strains and the complete genome of one selected strain demonstrated that all three patients had lineage-2 WNV infections. | Nagy et al., 2016 [85] |

WNV = West Nile virus, RT-PCR = reverse transcriptase polymerase chain reaction, ELISA = Enzyme linked immune assay, IF = immunofluorescence test, VI = virus isolation, NT= neutralisation test, PRNT = plaque reduction neutralisation test, IH = immunohistochemistry, TMA = transcription-mediated amplification, CSF = cerebrospinal fluid, NAT = nucleic acid amplification testing

**Table S5: Analysis of different sampling methods for diagnostic of Zika virus infection**

| **sample type** | **diagnostic methods** | **reason for analysis** | **results, key findings** | **reference** |
| --- | --- | --- | --- | --- |
| blood,  saliva, | RT-PCR | surveillance study of the out-break in Polynesia 2013/14 | ZIKV was more frequently detected in saliva compared to blood. For the 182 patients with both samples collected, tests were positive for 35 (19.2%) in saliva while negative in blood and tests were positive for 16 (8.8%) in blood while negative in saliva; the difference in mean days after symptoms onset and the percent-age of the main symptoms of Zika fever for patients only positive in saliva or in blood was not significant.  The use of saliva sample increased the rate of molecular detection of ZIKV at the acute phase of the disease but did not enlarge the window of detection of ZIKV RNA. Saliva was of particular interest when blood was difficult to collect (children and neonates especially). | Musso et al., 2015 [88] |
| plasma,  urine | RT-PCR,  IgG/IgM ELISA | diagnostic of an acute infection in returning travelers from West-Indies;  kinetics of virus load | ZIKV remained detectable in the plasma for roughly 2 weeks indicating that mosquito control measures should be prolonged accordingly. Remarkably, their urine samples consistently tested positive for even longer. The rapid onset of IgM allowed a diagnosis from the end of the first week. | Fourcade et al., 2016 [89] |
| serum,  urine,  saliva | RT-PCR | surveillance tracking of index case contacts in returning travelers to China | 9 imported Zika virus (ZIKV) infections (4 through temperature monitoring and epidemiological investigation at entry and five by active surveillance tracking of index case contacts during follow-up from Venezuela [n = 5], Samoa [n = 3] and both Samoa and Fiji [n = 1]) were detected in mainland China from Feb. 1- 29, 2016. The minimal incubation period lasted 5.2 days, with mean lag time to diagnosis of 2.6 days.  Diagnosis relied on positive RT-PCR for ZIKV RNA in serum (n = 7), urine (n = 4) or saliva (n = 3), respectively. | Zhang et al., 2016 [90] |
| plasma,  saliva,  urine, cerebrospinal fluid | RT-PCR  VI,  NT | research on pathogenesis in a rhesus macaque model of Asian-lineage Zika virus infection | Following subcutaneous inoculation, ZIKV RNA is detected in plasma 1 day post infection (d.p.i.) in all animals (N=8, including 2 pregnant animals), and is also present in saliva, urine and cerebrospinal fluid. Non-pregnant and pregnant animals remain viremic for 21 days and for up to at least 57 days, respectively. Neutralizing antibodies are detected by 21 d.p.i. | Dudley et al., 2016 [91] |
| semen,  blood,  urine,  saliva | RT-PCR,  VI | diagnostic of an acute infection in returning travelers to Korea | Zika virus was isolated from his semen, and RT- PCR was positive for the virus in the blood, urine, and saliva on the 7th day of the illness but was negative on the 21st day. | Jang et al., 2016 [92] |
| urine,  saliva | RT-PCR,  VI | diagnostic of acute infections;  analysis of non-vectorial ZIKV transmission routes | Nine urine and 5 saliva samples from nine patients from Rio de Janeiro presenting rash and other typical Zika acute phase symptoms were inoculated in Vero cell culture and submitted to specific ZIKV RNA detection and quantification through, respectively, NAT-Zika, RT-PCR and RT-qPCR. Two ZIKV isolates were achieved, one from urine and one from saliva specimens. | Bonaldo et al., 2016 [98] |
| urine,  saliva,  semen | RT-PCR | diagnostic of acute infections | Persistence of ZIKV RNA in blood, urine, saliva and semen was followed until the loads reached undetectable levels. RNA levels were higher in semen than in other sample types and declined to undetectable level at day 62 post onset of symptoms. | Reusken et al., 2016 [93] |
| saliva |  | review on routes for transmission | ZIKV is present in body fluids and has also been demonstrated in the saliva, but there is as yet no reliable evidence to support ZIKV transmission via this pathway | Leão et al., 2016 [100] |
| urine,  saliva,  serum | RT-PCR | diagnostic of acute infections | Urine specimens were collected from 70 patients with suspected Zika virus disease from 0 to 20 days after symptom onset. Of these, 65 (93%) tested positive for Zika virus RNA by RT-PCR. Results for 95% (52/55) of urine specimens collected from persons within 5 days of symptom onset tested positive by RT-PCR; only 56% (31/55) of serum specimens collected on the same date tested positive by RT-PCR. Results for 82% (9/11) of urine specimens collected >5 days after symptom onset tested positive by RT-PCR; none of the RT-PCR tests for serum specimens were positive. No cases had results that were exclusively positive by RT-PCR testing of saliva. BPHL testing results suggest urine might be the preferred specimen type to identify acute Zika virus disease. Rates of detection from urine were higher than from serum, even during the first few days after symptom onset and continuing after day five, when no serum specimens tested in this evaluation had detectable RNA. | Bingham et al., 2016 [97] |
| plasma,  saliva,  urine,  vaginal swab,  semen | RT-PCR,  VI | diagnostic of acute infection after sexual intercourse | Three days after the onset of her symptoms the urine sample of one patient tested positive for ZIKV RNA by RT-PCR at a viral count of 3.5×10^3^ copies/ml, and the saliva tested positive at a viral count of 2.1×10^4^ copies/ml. A plasma sample tested negative for ZIKV RNA by RT-PCR, but serum IgM antibodies to ZIKV were detected (see the [Supplementary Appendix](http://www.nejm.org/doi/suppl/10.1056/NEJMc1604449/suppl_file/nejmc1604449_appendix.pdf)). A vaginal swab obtained a week later was negative for ZIKV RNA by RT-PCR.  A urine sample from another patient obtained 16 days after the onset of symptoms tested positive for ZIKV RNA by RT-PCR with a viral count of 4×10^3^ copies/ml, but plasma and saliva samples tested negative. The first and second urine stream samples obtained on day 24 were positive for ZIKV RNA with a viral count of 2.1×10^4^ copies/ml. Semen samples tested positive for ZIKV RNA by RT-PCR with a high viral load of 2.9×10^8^ copies/ml in the sample obtained on day 18 and 3.5×10^7^ copies/ml in the sample obtained on day 24. ZIKV was isolated by means of culture from semen samples on days 18 and 24. | D'Ortenzio et al., 2016 [94] |
| blood,  saliva,  urine | RT-PCR,  IFA,  ELISA | diagnostic of acute infection after returning from Dominican Rep. | The patient had prolonged shedding of viral RNA in saliva and urine, at higher load than in blood, for up to 29 days after symptom onset. | Barzon et al., 2016 [96] |
| serum,  urine | RT-PCR | diagnostic of an acute infection after sexual intercourse | Zika virus RNA was detected in both serum and urine by RT-PCR | Davidson et al., 2016 [95] |
| amniotic fluid,  breast milk, semen, saliva,  urine,  blood | RT-PCR | reviewing non-mosquito-borne transmission routes | Our systematic analysis shows that non-vector-borne ZIKV transmission plays a role in the spread of ZIKV and has great societal impact. It has important public health implications for the prevention and control of ZIKV globally and will be a basis for policy and further research. | Grischott et al., 2016 [99] |
| blood,  urine,  saliva, amniotic fluid,  body fluids |  | analysis/recommendation of safety measures for health care workers | Zika virus RNA has been detected in a number of body fluids, including blood, urine, saliva, and amniotic fluid (3-5), and whereas transmission associated with occupational exposure to these body fluids is theoretically possible, it has not been documented. Although there are no reports of transmission of Zika virus from infected patients to health care personnel or other patients, minimizing exposures to body fluids is important to reduce the possibility of such transmission. CDC recommends Standard Precautions in all health care settings to protect both health care personnel and patients from infection with Zika virus as well as from blood-borne pathogens (e.g., human immunodeficiency virus [HIV] and hepatitis C virus [HCV]) | Olson et al., 2016 [101] |
| saliva |  | analysis/recommendation of safety measures for health care workers (dentists) | In the end the, we demonstrated the importance of a more comprehensive exploration of saliva and their components as a fluid for diagnostic and therapeutic approaches on oral and systemic diseases | Siqueira et al., 2016 [103] |
| saliva,  urine |  | analysis/recommendation of safety measures for health care workers | This article will discuss how healthcare workers could contract diseases especially whilst working in the tropics or subtropics due to disease vectors such as mosquitoes and suggests prevention measures for this group. | Scully et al., 2016 [102] |

NT = neutralization test, IFA = immunofluorescence assay, ELISA = enzyme-linked immunosorbent assay, RT-PCR= Reverse transcription polymerase chain reaction, VI = virus isolation

**Table S6: Analysis of different sampling methods for diagnostic of Yellow Fever virus infection**

| **sample type** | **diagnostic methods** | **reason for analysis** | **results, key findings** | **reference** |
| --- | --- | --- | --- | --- |
| urine | RT-PCR | surveillance study for analysis of adverse events in vaccinees | In 18 of 129 urine samples yielded positive amplification of the YF-17D RNA, while all pre-immune samples were negative. Among the healthy YF-17D vaccinees, 4 exhibited the presence of YFV RNA in their urine (28.6%), including 3 first-time vaccinees and the revaccinated one. The YF-17D genome was detected in the urine of these individuals in an intermittent mode, with more than one consecutive day yielding positive amplification. It seems that a 1st excretion of YF-17D occurs in the first days after vaccination and a 2nd viral shedding (days 4 to 7) might happen, probably reflecting the viral replication in the vaccinees. Among suspected YFVAE patients, YF-17D RNA was detected in 8 of 18 patients (44.4%) at different time points. Paired sera from these patients did not yield a positive amplification of YFV-17D RNA. Remarkably, we found the presence of viral RNA 20, 24, and 25 days after vaccination in the suspected YFVAE patients. We can only hypothesize whether the presence of viral RNA in urine at this time was a response to the prolonged replication of the virus in the patients affected or to a persistent viral shedding of the vaccine virus which may occur in some individuals without further pathological significance. The average viral load detected in positive samples was 8.8 10^2^ GE/ml, ranging between 30 and 70 GE/ml (n = 4 samples) to 10^4^ GE/ml (n = 1 sample), without any significant difference regarding the day of sample collection. | Domingo et al., 2011 [104] |
| urine | RT-PCR | surveillance study for chronic persistent infection in vaccinees | Urine samples from 44 healthy recipients of yellow fever vaccine at varying times up to one year after vaccination were tested. Urine samples from two vaccine recipients had detectable yellow fever virus RNA. The time since vaccination was reported as 21 days for 1 sample and 198 days for the other sample. These results suggest that 17D YF vaccine virus might persist for at least 6 months after vaccination in some people. | Martínez et al., 2011 [105] |

GE = genome equivalent, YF = Yellow Fever, AE = adverse event

**Table S7: Analysis of different sampling methods for diagnostic of severe acute respiratory syndrome (SARS) virus infection**

| **sample type** | **diagnostic methods** | **reason for analysis** | **results, key findings** | **reference** |
| --- | --- | --- | --- | --- |
| NPA,  blood,  urine,  faecal | RT-PCR,  AG-ELISA, | surveillance study on pathogenesis and evaluation of Ribavirin treatment | RT-PCR on respiratory and faecal samples, together with serology, can confirm the diagnosis of SARS-associated coronavirus infection in most SARS patients. IgG seroconversion was documented in 70 (93%) patients at mean of 20 (5.1) days. SARS-associated coronavirus RNA was detected in NPA by RT-PCR in 24 (32%) of 75 patients at initial presentation (mean 3.2 [1.3] days after onset) and in 51 (68%) at day 14. In stool samples collected later in the illness (a mean of 14.2 [2.2] days after onset), viral RNA was detected in 65 (97%) of 67. Similarly, viral RNA was detected in 31 (42%) of 74 urine samples collected at a mean of 15.2 (1.7) days after onset of symptoms. | Peiris et al., 2003 [106] |
| throat wash,  saliva | RT-PCR,  IF | diagnostic of acute infection | We examined oral specimens, including throat wash and saliva, and found large amounts of SARS-CoV RNA in both throat wash 9.6 x 10^2^ to 6.0 x 10^6^ copies/ml and saliva 7.1 x 10^3^ to 6.4 x 10^8^ copies/ml from all specimens of 17 consecutive probable SARS case-patients, supporting the possibility of transmission through oral droplets.  Immunofluorescence study showed replication of SARS-CoV in the cells derived from throat wash, demonstrating the possibility of developing a convenient antigen detection assay. This finding, with the high detection rate a median of 4 days after disease onset and before the development of lung lesions in four patients, suggests that throat wash and saliva should be included in sample collection guidelines for SARS diagnosis. | Wang et al., 2004 [107] |
| plasma,  sputum, endotracheal aspirates,  stool,  throat swabs, saliva | RT-PCR,  IF | diagnostic of acute infection | In clinical samples the median concentrations of R- and N-gene RNA, respectively, were 1.2 x 10^6^ and 2.8 x 10^6^ copies/ml (sputum and endotracheal aspirates), 4.3 x 10^4^ and 5.5 x 10^4^ copies/ml (stool), and 5.5 x 10^2^  and 5.2 x 10^2^ copies/sample (throat swabs and saliva). Differences between the samples types were significant but not between the types of target RNA. All (n = 12) samples from the lower respiratory tract tested positive in all tests. Methods for the routine sampling of sputum without infection risk are needed to improve SARS RT-PCR. | Drosten et al., 2004 [108] |
| NPA,  serum,  stool,  urine | RT-PCR,  VI,  IF | surveillance on the pathogenesis of acute infection | A higher mean virus load in nasopharyngeal specimens obtained on day 10 after the onset of symptoms was significantly associated with the occurrence of diarrhea (3.1 log10 vs. 1.8 log10 copies/mL; ) and mortality (6.2 vs. 1.7 log10 Pp.01 copies/mL; P ! .01). However, diarrhea was not associated with mortality. The lung and the gastrointestinal tract may react differently to SARS coronavirus infection. Additional investigation of the role of SARS coronavirus in the pathogenesis of diarrhea in patients with SARS should be conducted. | Cheng et al., 2004 [109] |
| NPA,  serum,  stool,  urine | RT-PCR,  VI,  IF | surveillance on the pathogenesis of acute infection | The importance of SARS-CoV as a respiratory pathogen is supported by the strong association of viral load in NPA with oxygen desaturation, mechanical ventilation, and death. Unexpectedly, viral load in NPA was also associated with diarrhea and hepatic dysfunction. High viral load in urine is also associated with abnormal urinalysis findings. In fact, viral load in stool is also associated with death. High viral load in NPA, with or without high viral load in serum, is a useful prognostic indicator of respiratory failure or death. The presence of viral RNA in multiple body sites also indicates poor prognosis. | Hung et al., 2004 [112] |
| NPA | RT-PCR | surveillance on the pathogenesis of acute infection | The initial results of qPCR of the nasopharyngeal specimens ranged from undetectable to 8.8 log 10 RNA copies/ml. There was no correlation between the viral load and the number of days elapsed from symptom onset to presentation. | Chu et al., 2004 [111] |
| throat swabs, nasal swabs, rectal swab, NPA,  urine,  stool | RT-PCR | study on different diagnostic samples of patients with acute infection | Virological test results of 415 patients with severe acute respiratory syndrome (SARS) were examined. The peak detection rate for SARS-associated coronavirus occurred at week 2 after illness onset for respiratory specimens, at weeks 2 to 3 for stool or rectal swab specimens, and at week 4 for urine specimens. The latest stool sample that was positive by RT-PCR was collected on day 75 while the patient was receiving intensive care. Tracheal aspirate and stool samples had a higher diagnostic yield (RT-PCR average positive rate for first 2 weeks: 66.7% and 56.5%, respectively). Pooled throat and nasal swabs, rectal swab, nasal swab, throat swab, and nasopharyngeal aspirate specimens provided a moderate yield (29.7%-40.0%), whereas throat washing and urine specimens showed a lower yield (17.3% and 4.5%). The collection procedures for stool and pooled nasal and throat swab specimens were the least likely to transmit infection, and the comb-ination gave the highest yield for coronavirus detection by RT-PCR. | Chan et al., 2004 [110] |
| NPA,  stool,  rectal swaps,  urine | RT-PCR | evaluation of commercial PCR kits for diagnostic quality | Testing 68 clinical specimens (including 17 respiratory tract specimens, 29 urine samples, and 22 stools or rectal swabs) demonstrated that six of the seven assays detected at least 17 of 18 positives and two of the assays had a sensitivity of 100%. | Mahony et al., 2004 [114] |
| NPA | RT-PCR | surveillance on the transmission of acute infections | Higher nasopharyngeal viral load was found in patients living in adjacent units of the same block inhabited by the index patient, while a lower but detectable nasopharyngeal viral load was found in patients living further away from the index patient. This pattern of viral load in NPA suggested that airborne transmission played an important part in this outbreak in Hong Kong. | Chu et al., 2005 [113] |
| nasopharyngeal swab,  rectal swab,  tracheal aspirate,  stool,  urine | RT-PCR, | diagnostic of acute infection | Varying amounts of SARS-CoV were found in the 26 SARS-CoV positive specimens and SARS-CoV was not detected in the 40 follow up specimens and controls. Finally, SARS-CoV and GAPDH mRNA were both detected in three out of eight follow up urine specimens that were initially negative when the amount of cDNA used was increased. Moreover, GAPDH mRNA may be useful to rule out false negative results in SARS-CoV detection, and the current extraction method for urine may not be sensitive enough to detect low titres of SARS-CoV. This area demands further investigation. | Wong et al., 2005 [116] |
| serum,  fecal,  urine | RT-PCR,  IF,  AG-ELISA | evaluation of in-house AG-ELISA for diagnostic quality | The method of choice for early diagnosis of SARSCoV infection should be the RT-PCR. The sensitivity of RT-PCR is superior to that of both ELISAs. Moreover, RT-PCR can detect SARS-CoV earlier in fecal specimens. Among the 40 fecal samples from SARS patients, 32 (80%) were positive by RT-PCR, which was significantly higher than that of the antibody-based ELISAs. Of the 133 urine samples from SARS patients, 33 (25%) were positive by RT-PCR, which was also significantly higher than that of antibody-based ELISAs. When RT-PCR was used as a standard, the sensitivities of the ELISAs were 53.1% (17/32) and 43.8% (14/32) in fecal specimens, and 12.1% (4/33) and 15.2% (5/33) in urine specimens, respectively. The RT-PCR can detect SARS-CoV in fecal specimens obtained on days 1 to 27 after onset of symptoms and in urine specimens obtained on days 9 to 45. Moreover, 6 (75%) of the 8 fecal specimens obtained on days 1 to 10 were positive by RT-PCR. All 3 tests had the highest detection rates in fecal specimens collected on days 16 to 20, which suggested that this was the period of peak viral shedding in stool. The detection rates in urine specimens were much lower than those in fecal specimens in all 3 assays. | Lau et al., 2005 [115] |
| serum,  plasma,  urine,  stool | RT-PCR,  IgG-ELISA | surveillance on the pathogenesis of infections | Data showed that 67 of the 73 SARS patients demonstrated sero-conversion by week 5 of illness. In contrast, only 1 of 278 healthy subjects enrolled in the study was found to be positive for the IgG antibody. Coexistence of viral RNA in plasma and specific antibodies was simultaneously observed over three consecutive weeks in two critical cases. In three convalescent patients in particular, cultivable SARS-CoV was detected in stool or urine specimens for longer than 4 weeks (29-36 days). These findings suggest that SARS-CoV may remain viable in the excretions of convalescent patients. | Xu et al., 2005 [117] |
| urine,  stool | semi-nested  RT-PCR | surveillance study for virus transmission via sewage of hospitals | This study demonstrated that there was SARS-CoV RNA in stool samples of patients with symptoms and in sewage of hospitals though there was no live SARSCoV isolated from all samples. It provides evidence that the nucleic acid of SARS-CoV can be excreted through the stools of patients into sewage system, but cannot exclude the possibility of SARS-CoV transmitting through the digestive system. | Wang et al., 2005 [118] |
| NPA ,  throat-swab,  fecal , cerebrospinal fluid,  blood,  urine | RT-PCR,  VI | recommendation for the diagnostic of acute respiratory infections | The detection of SARS virus is possible in various clinical samples (including urine) by viral culture or RT-PCR. Clinical samples needed for a specific diagnosis are: nasopharyngeal, throat-swab or faecal samples, cerebrospinal fluid and blood. The presence of A/H5N1 virus is confirmed by viral isolation or RNA detection by RT-PCR. | Goffard et al., 2006 [119] |
| fecal,  oral,  urine,  tissue samples | meta-genomic analysis | surveillance study in three common North American bat species for viral pathogens | In samples derived from these three bat species, we identified viral sequences that were similar to at least three novel groups 1 CoVs, large numbers of insect and plant virus sequences, and nearly full-length genomic sequences of two novel bacteriophages. These observations suggest that bats encounter and disseminate a large assortment of viruses capable of infecting many different animals, insects, and plants in nature. | Donaldson et al., 2010 [121] |
| throat swabs | ELISA IgG, IgA | research on mucosal immunity in mice | Our study indicates that mucosal immunization with rBV SARS-CoV Virus like particles represent an effective means for eliciting protective systemic and mucosal immune responses against SARS-CoV, providing important information for vaccine design. | Lu et al., 2010 [123] |
| salivary glands | RT-PCR,  IH | research on pathogenesis in rhesus macaques | Our findings provide evidence that salivary gland epithelial cells can be infected in vivo soon after infection. These infected cells could be a significant source of virus in saliva, particularly early in infection. This observation has significant implications for understanding SARS-CoV respiratory transmission, which is critical for the development of effective strategies for diagnosis, prevention, and therapy. | Liu et al., 2011 [122] |

NPA = nasopharyngeal aspirates, AG-ELISA = antigen capture ELISA, RT-PCR = reverse transcriptase PCR, IF = immuno fluorescence, VI = virus isolation, IH = immuno histology,

**Table S8: Analysis of different sampling methods for diagnostic of Middle East respiratory syndrome coronavirus (MERS-CoV) infection**

| **sample type** | **diagnostic methods** | **reason for analysis** | **results, key findings** | **reference** |
| --- | --- | --- | --- | --- |
| bronchial-veolar fluid, blood,  urine,  oronasal swabs | RT-PCR,  IFA | analyzing clinical features and viremia in acute case | MERS-CoV was detected in 2 samples of bronchoalveolar fluid. Viral loads were highest in samples from the lower respiratory tract (up to 1·2 × 10^6^ copies per mL). Maximum virus concentration in urine samples was 2691 RNA copies per mL on day 13; the virus was not present in the urine after renal failure on day 14. Stool samples obtained on days 12 and 16 contained the virus, with up to 1031 RNA copies per g (close to the lowest detection limit of the assay). One of 2 oronasal swabs obtained on day 16 were positive, but yielded little viral RNA (5370 copies per mL). No virus was detected in blood. | Drosten et al., 2013 [124] |
| kidney tissue | RT-PCR,  VI | review differences in descriptions of kidney involvement in MERS-CoV versus SARS patients | Epidemiological studies should analyze kidney impairment and its characteristics in MERS-CoV. Virus replication in the kidney with potential shedding in urine might constitute a way of transmission, and could explain untraceable transmission chains leading to new cases. Individual patients might benefit from early induction of renoprotective treatment. | Eckerle. et al., 2013 [125] |
| urine,  saliva,  fecal |  | evaluate a prediction model for oral-fecal and or oral-saliva transmission routes for MERS-CoV | Oral-urine and saliva transmission are also highly possible since both require harder protective shells. Results show that disorder prediction can be used as a tool that suggests clues to look for in further epidemiological investigations. | Goh et al., 2013 [126] |
| bronchial-veolar fluid, exudate mouth,  exudate nose,  stool,  urine | RT-PCR | evaluation of a commercial RT-PCR kit for diagnostic | The performance of both RT-PCR assays included in the kit is comparable to the in-house assays. They show high analytical sensitivity, no cross-reactivity with other respiratory pathogens and detected MERS-CoV RNA in patient samples in almost the same manner as the in-house versions. | Corman et al., 2014 [128] |
| blood,  urine,  saliva,  fecal | RT-PCR,  sequenc-ing,  VI,  IFA,  NT | analysis of European hedgehogs for CoVs by broad-range nested RT-PCRs | A total of 58.9% of hedgehog fecal specimens were positive for the novel CoV (EriCoV) at 7.9 log10 mean RNA copies per ml. EriCoV RNA concentrations were higher in the intestine than in other solid organs, blood, or urine. Detailed analyses of the full hedgehog intestine showed the highest CoV concentrations in European hedgehogs in lower gastrointestinal tract specimens, compatible with viral replication in the lower intestine and fecal-oral transmission. 13 of 27 (48.2%) hedgehog sera contained non-neutralizing antibodies against MERS-CoV. The animal origins of this beta-coronavirus clade that includes MERS-CoV may thus include both bat and non-bat hosts. | Corman et al., 2014 [137 ] |
| blood,  urine,  nasal swab,  pharyngeal swab,  tracheal swab,  rectal swab | RT-PCR | analyse the kinetics and pattern of viral excretion in 2 infected patients | In patient 1, who died of refractory acute respiratory distress syndrome and renal failure, MERS-CoV RNA was detected in pharyngeal and tracheal swabs, as well blood samples and urine samples until the 30^th^ day. Rectal swabs were negative. Patient 2 also developed multiple-organ failure, but survived, with persisting renal insufficiency and persistent interstitial syndrome. Tracheal aspirations were positive until the 33^rd^ day, while nasopharyngeal swabs were negative. All other biological samples were negative. LR tract excretion of MERS-CoV could be observed for more than one month. The most severely ill patient presented an expression of the virus in blood and urine, consistent with a type-1 interferon mediated immunological response impaired in patient 1, but developed by patient 2. These results suggest that infection control precautions must be adequately evaluated in clinical wards and laboratories exposed to MERS-CoV. | Poissy et al., 2014 [138] |
| nasal swaps,  fecal swaps,  camel milk | RT-PCR,  ELISA | review analyzing the transmission between camels, bat and human | The seroprevalence of MERS-CoV antibodies is very high in dromedary camels in Eastern Africa and the Arabian Peninsula. MERS-CoV RNA and viable virus have been isolated from dromedary camels, including some with respiratory symptoms. | Omrani et al., 2015 [127] |
| blood,  urine,  sputum,  URS,  LRS | RT-PCR | analysis of acute case during an MersCoV outbreak in Korea | 30 MERS-CoV patients admitted to the Na­tional Medical Center, Korea from May to July 2015. We followed up with each patient until expiration or discharge from the hos­pital. Half of the patients in the present study showed proteinuria, and more than one-fourth of the patients devel­oped acute kidney injury. Further studies are needed to reveal the direct effects of MERS-CoV on renal complications. | Cha et al., 2015 [129] |
| LRS | RT-PCR | analyzing the infectivity of MersCoV | Current data indicate that the virus spreads from human to human through droplet and contact routes, while performing aerosole-generating procedures predispose to airborne transmission. The best diagnostic tests rely on the identification of MERS-CoV by PCR, and lower respiratory tract samples should be favoured for the diagnosis whenever this is possible in order to avoid false negative results. Recently, the World Health Organization added serology to the list of confirmatory tests. | Al-Tawfiq, Memish, 2015 [130] |
| saliva | RT-PCR,  LAMP | evaluating the LAMP method for diagnosis of MersCoV genome | Combining this high temperature isothermal amplification method with a thermostable invertase, we can directly transduce MersCoV and Zaire Ebolavirus templates into glucose signals, with a sensitivity as low as 20–100 copies/µ l. Virus from cell lysates and synthetic templates could be readily amplified and detected even in sputum or saliva. The method describes has potential for accelerating point-of-care applications, in that biological samples could be applied to a transducer that would then directly interface with an off-the-shelf, approved medical device. | Du et al., 2015 [133] |
| blood,  stool,  urine  LRS,  URS | RT-PCR,  IFA,  VN | analyzing viral shedding and antibody response in 37 MERS-CoV patients | The timing and intensity of respiratory viral shedding in patients with MERS closely matches that of those with severe acute respiratory syndrome. Blood viral RNA does not seem to be infectious. Extra-pulmonary loci of virus replication seem possible. Neutralizing antibodies are not sufficient to clear the infection. | Corman et al., 2016 [131] |
| camel milk,  meat,  urine |  | review analyzing the transmission between camels and human | MERS-CoV) cases without documented contact with another human MERS-CoV case make up 61% (517/853) of all reported cases. These primary cases are of particular interest for understanding the  source(s) and route(s) of transmission and for designing long-term disease control measures. Dromedary camels are the only animal species for which there is convincing evidence that it is a host species for MERS-CoV and hence a potential source of human infections. However, only a small proportion of the primary cases have reported contact with camels. Further microbiological experiments on milk, meat and urine should urgently be conducted to establish whether they are (effective) vehicles for virus transmission. | Gossner et al., 2016 [132] |

URS = upper respiratory speci­mens, LRS = lower respiratory speci­mens, NT, = virus neutralizing test, VI = virus isolation, LAMP = loop-mediated

isothermal amplification

**Table S9: List of publications describing samples used for viral diagnostic. The number in brackets referred to the reference.**

| **Type of specimens** | | **Arboviruses** | | | | | **VHFV** | **Coronaviruses** | |
| --- | --- | --- | --- | --- | --- | --- | --- | --- | --- |
|  |  | **CHIKV** | **DENV** | **WNV** | **YFV** | **ZIKV** | **EBOV** | **SARS** | **MERS** |
| **Blood derived samples** | **whole blood** | [139] | [143] | [147,148] | n.r. | [88] | [160] | [165] | [170] |
|  | **peripheral blood (cells)** | [139] | [144] | [147,148] | n.r. | n.r. | [54] | [166] |  |
|  | **peripheral blood (plasma)** | [140] | [13] | [149] | [152] | [89] | [54] | [112,166] |  |
|  | **serum*** | [141] | [144] | [149] | [152] | [97] | [52] | [167] | [169] |
|  | **umbilical cord blood** | n.r. | n.r. | n.r. | n.r. | n.r. | n.r. | n.r. | n.r. |
| **Respiratory samples** | **nasopharyngeal swab** | n.r. | n.r. | n.r. | n.r. | n.r. | n.r. | [110,111] | [124] |
|  | **oropharyngeal swab** | n.r. | n.r. | n.r. | n.r. | n.r. | n.r. | [110] | [124] |
|  | **bronchoalveolar lavage** | n.r. | n.r. | n.r. | n.r. | n.r. | n.r. | [110] | [171,124] |
|  | **sputum** | n.r. | n.r. | n.r. | n.r. | n.r. | n.r. | [169] | [171] |
|  | **saliva** | [19] | [13] | n.r. | ? | [88,89,97] | [52] | n.r. | n.r. |
| **Body fluids** | **sweat** | n.r. | n.r. | n.r. | n.r. | [157] | [54] | n.r. | n.r. |
|  | **tears** | n.r. | n.r. | n.r. | n.r. | [157] | [53] | n.r. | n.r. |
|  | **cerebrospinal fluid** | [142] | [145] | [150] | [154] | [156] | [163] | [168] |  |
|  | **urine** | [19] | [19] | [75] | [104] | [88,89,97] | [54] | [110,112] | [124] |
|  | **vaginal discharge** | n.r. | n.r. | n.r. | n.r. | [94] | [164] | n.r. | n.r. |
|  | **seminal fluid** | n.r. | n.r. | n.r. | n.r. | [94] | [161,53] | n.r. | n.r. |
| **Other specimens** | **feces** | n.r. | n.r. | n.r. | n.r. | n.r. | [53] | [110,112] | [124] |
|  | **amniotic fluid** | n.r. | n.r. | n.r. | n.r. | [158] | [162] | n.r. | n.r. |
|  | **placenta** | n.r. | n.r. | n.r. | n.r. | [155] | [162] | n.r. | n.r. |
|  | **breast milk** | n.r. | [146] | [151] | [153] | [159] | [53] | n.r. | n.r. |

VHF = Viral haemorrhagic Fever, * = NAT & Immunological test, n.r. = not reported
